# Inflammasomes in Alveolar Bone Loss

**DOI:** 10.3389/fimmu.2021.691013

**Published:** 2021-06-09

**Authors:** Yang Li, Junqi Ling, Qianzhou Jiang

**Affiliations:** ^1^ Department of Endodontics, Affiliated Stomatology Hospital of Guangzhou Medical University, Guangzhou Key Laboratory of Basic and Applied Research of Oral Regenerative Medicine, Guangzhou, China; ^2^ Guangdong Province Key Laboratory of Stomatology, Department of Operative Dentistry and Endodontics, Guanghua School of Stomatology, Hospital of Stomatology, Sun Yat-sen University, Guangzhou, China

**Keywords:** alveolar bone loss, bone remodeling, inflammasome, inflammation, interleukin-1, osteolysis, pyroptosis, periodontitis

## Abstract

Bone remodeling is tightly controlled by osteoclast-mediated bone resorption and osteoblast-mediated bone formation. Fine tuning of the osteoclast–osteoblast balance results in strict synchronization of bone resorption and formation, which maintains structural integrity and bone tissue homeostasis; in contrast, dysregulated bone remodeling may cause pathological osteolysis, in which inflammation plays a vital role in promoting bone destruction. The alveolar bone presents high turnover rate, complex associations with the tooth and periodontium, and susceptibility to oral pathogenic insults and mechanical stress, which enhance its complexity in host defense and bone remodeling. Alveolar bone loss is also involved in systemic bone destruction and is affected by medication or systemic pathological factors. Therefore, it is essential to investigate the osteoimmunological mechanisms involved in the dysregulation of alveolar bone remodeling. The inflammasome is a supramolecular protein complex assembled in response to pattern recognition receptors and damage-associated molecular patterns, leading to the maturation and secretion of pro-inflammatory cytokines and activation of inflammatory responses. Pyroptosis downstream of inflammasome activation also facilitates the clearance of intracellular pathogens and irritants. However, inadequate or excessive activity of the inflammasome may allow for persistent infection and infection spreading or uncontrolled destruction of the alveolar bone, as commonly observed in periodontitis, periapical periodontitis, peri-implantitis, orthodontic tooth movement, medication-related osteonecrosis of the jaw, nonsterile or sterile osteomyelitis of the jaw, and osteoporosis. In this review, we present a framework for understanding the role and mechanism of canonical and noncanonical inflammasomes in the pathogenesis and development of etiologically diverse diseases associated with alveolar bone loss. Inappropriate inflammasome activation may drive alveolar osteolysis by regulating cellular players, including osteoclasts, osteoblasts, osteocytes, periodontal ligament cells, macrophages, monocytes, neutrophils, and adaptive immune cells, such as T helper 17 cells, causing increased osteoclast activity, decreased osteoblast activity, and enhanced periodontium inflammation by creating a pro-inflammatory milieu in a context- and cell type-dependent manner. We also discuss promising therapeutic strategies targeting inappropriate inflammasome activity in the treatment of alveolar bone loss. Novel strategies for inhibiting inflammasome signaling may facilitate the development of versatile drugs that carefully balance the beneficial contributions of inflammasomes to host defense.

## Introduction

The alveolar bone, an important part of the maxillofacial skeleton, is a connective tissue that supports teeth, is subjected to mechanical stress, and undergoes continuous bone remodeling (1). Similar to other bone tissues, osteoclasts and osteoblasts are the main components responsible for the highly dynamic equilibrium between bone resorption and formation in the alveolar bone. In addition to these two vital players, a complex cellular communication network, including osteocytes, macrophages, monocytes, neutrophils, and adaptive immune cells, such as T helper 17 cells (Th17 cells), also plays critical roles in maintaining strict bone coupling and alveolar bone homeostasis (2). Alveolar bone remodeling is not only a part of the bone turnover of the skeletal system but also mirrors skeletal bone conditions. Interestingly, the turnover rate of alveolar bone is significantly higher in the mandible and maxilla than in the femur and at the alveolar crest than at the level of the mandibular canal and the inferior compact border, suggesting highly dynamic remodeling of the alveolar bone (3, 4). The association between the tooth and periodontium also increases the complexity of alveolar bone remodeling. Pathogen invasion from the oral environment or hematogenous spread, mechanical stress from orthodontic treatments, medication, and systemic pathological factors can induce sophisticated inflammation, which dictates the activities of osteoclasts and osteoblasts in alveolar bone, shifting the balance of bone homeostasis to increase bone resorption and decrease bone formation. Hence, it is important to understand the osteoimmunological mechanism of alveolar bone loss.

Innate immunity acts as the front line of defense against pathogen invasion and tissue damage. Inflammasomes serve as intracellular pattern recognition receptors to activate inflammatory caspases (5). In response to pathogen-associated molecular patterns (PAMPs) and damage-associated molecular patterns (DAMPs), canonical inflammasomes are activated as a multimolecular protein complex and platform to recruit caspase-1, leading to its autoproteolytic activation, subsequent production of mature interleukin (IL)-1β and IL-18, and a lytic form of cell death called pyroptosis. Likewise, noncanonical inflammasome caspases (human caspase-4/5 and mouse caspase-11) act as both the sensor and effector, recognize stimuli such as intracellular lipopolysaccharide (LPS), and induce pyroptosis (6). The inflammasome is not only a key regulator of innate immunity but also plays critical roles in adaptive immunity, making it a pivotal player in the immune response and host defense (7). Appropriate inflammasome activity is required for wound healing and bone homeostasis, whereas inappropriate inflammasome activity could negatively influence host defense and homeostasis (8). Pathogens and their by-products may inhibit inflammasome activation to escape host immune defense, resulting in persistent infection or spreading of infection (9). However, excessive inflammasome activity can contribute to the pathogenesis and development of various diseases associated with bone destruction (10–13). Hence, inflammasomes act as a double-edged sword with both protective and detrimental potential for host defense and bone remodeling. Inflammasomes also play critical roles in unbalanced alveolar bone remodeling, which may occur as a local dysregulation or as part of systemic bone diseases. A comprehensive understanding of the mechanisms of inflammasomes in alveolar bone loss may contribute to the identification of therapeutic targets and the development of novel anti-inflammatory drugs.

Here, we review the recent advancements and insights into the potential mechanisms of inflammasomes in the pathogenesis and development of alveolar bone loss and discuss the potential and novel therapeutic strategies targeting inappropriate inflammasome activity in this field.

## Structure and Activation of Inflammasomes

The inflammasome, an intracellular supramolecular protein complex, is activated upon sensing PAMPs and DAMPs. Among the canonical inflammasomes, nucleotide-binding oligomerization domain (NOD)-like receptors (NLRs), absent in melanoma 2 (AIM2)-like receptors (ALRs), and pyrin play pivotal roles in the innate immune response (14). Noncanonical inflammasomes induce pyroptosis and secondary activation of other inflammasomes (15). Inflammasomes can be modulated by several regulators, such as caspase activation and recruitment domain (CARD)-only proteins, pyrin domain (PYD)-only proteins, interferons (IFNs), autophagy molecules, and tripartite motif (TRIM) proteins, which have been reviewed elsewhere (16–18). Herein, we focus on the structure and activation of canonical and noncanonical inflammasomes (**Table 1**).

**Table 1:** Molecules or domains associated with inflammasome activation.

Name	Main functions in inflammasome activation
Absent in melanoma 2 (AIM2)	AIM2 functions as a canonical inflammasome for DNA recognition (19–21).
AIM2-like receptors (ALR)	Four and 13 ALRs are expressed in humans and mice, respectively; only AIM2 and IFI16 function as inflammasomes for the recognition of cytoplasmic and nuclear DNA from pathogens and damaged cells (14).
Apoptosis associated speck-like protein containing a CARD (ASC)	ASC binds to inflammasomes and caspase-1 *via* the homotypic interaction of PYD-PYD and CARD-CARD, respectively (8).
Baculoviral IAP repeat-containing proteins (NAIPs)	NAIPs in NLR family have three baculovirus inhibitor-of-apoptosis repeats at the N-terminus. Humans express only one NAIP, which recognizes the T3SS needle protein of bacteria such as *Chromobacterium violaceum*. Recognition of flagellin by NAIP5 and NAIP6 as well as recognition of T3SS proteins by NAIP2 indirectly activate caspase-1 through NLRC4 oligomerization (22).
Canonical inflammasomes	Canonical inflammasomes are activated as a multimolecular protein complex and platform to recruit caspase-1, leading to its autoproteolytic activation, subsequent production of mature IL-1β and IL-18, and pyroptosis (14).
Caspase activation and recruitment domain (CARD)	CARD is a domain in inflammasomes that directly binds to its counterpart domain in caspase-1 for its recruitment (23).
Caspase-1	Caspase-1 is recruited by canonical inflammasomes, leading to its autoproteolytic activation, subsequent production of mature IL-1β and IL-18, and pyroptosis (23).
Caspase-4	Caspase-4 in humans can convert GSDMD into GSDMD-N to induce pyroptosis. Caspase-4 may process pro-IL-18 but not pro-IL-1β (24).
Caspase-5	Caspase-5 in humans can convert GSDMD into GSDMD-N to induce pyroptosis. Caspase-5 possesses a weak ability to process pro-IL-1β and pro-IL-18 (24).
Caspase-11	Caspase-11 in mice can convert GSDMD into GSDMD-N to induce pyroptosis. Caspase-11 is not able to process pro-IL-1β and pro-IL-18 (24).
Conserved in UNC5, PIDD, and ankyrin domain (UPA)	UPA is a domain in FIIND of NLRP1. FIIND autoprocessing yields two polypeptides: UPA-CARD and NACHT-LRR-ZU5 (25).
Cyclic GMP-AMP synthase (cGAS)/stimulator of IFN genes (STING)/STING-TANK binding kinase 1 (TBK1)/IRF3 axis	This axis drives IFN regulatory factor 1 (IRF1) expression upon which GBP2/GBP5 and IRGB10 are produced (26–28).
Damage-associated molecular patterns (DAMPs)	DAMPs are associated with host damage and endogenous danger signals (e.g., extracellular heat shock protein 70) (5).
Found in ZO-1 and UNC5 domains (ZU5)	ZU5 is a domain in FIIND of NLRP1. FIIND autoprocessing yields two polypeptides: UPA-CARD and NACHT-LRR-ZU5 (25).
Function-to-find domain (FIIND)	FIIND is a domain in NLRP1 that may undergo autoprocessing (25).
Gasdermin D (GSDMD)	GSDMD can be cleaved by caspase-1/-4/-5/-11 to induce pyroptosis (29).
GSDMD N-terminal fragment (GSDMD-N)	GSDMD-N interacts with the inner membrane glycerophospholipids of the lipid bilayer, forming pores on cell membranes and triggering pyroptosis (29).
Guanylate-binding protein 2 (GBP2) and protein 5 (GBP5)	GBP2 and GBP5 disrupt the bacterial membrane and vacuoles containing bacteria, leading to bacteria and DNA exposure (26, 28).
IFN-gamma inducible 16 (IFI16)	IFI16 is a canonical inflammasome in ALR family. It is located in the nucleus, has two HIN-200 domains, and forms an inflammasome upon infection by viruses such as herpesviruses (30).
Immunity-related GTPase family member b10 (IRGB10)	IRGB10 disrupts the bacterial membrane and vacuoles containing bacteria, leading to bacteria and DNA exposure (26, 28).
Interleukin (IL)-1β	Caspase-1 can process pro-IL-1β into IL-1β during inflammasome activation (9).
Interleukin (IL)-18	Caspase-1 can process pro-IL-18 into IL-18 during inflammasome activation (9).
Leucine-rich repeat (LRR)	LRR is a domain in NLRs that contributes to ligand recognition and post-translational modifications (9).
NIMA-related kinase 7 (NEK7)	NEK7 interacts with LRR and NBD in NLRP3 to promote NLRP3 activation (31).
NLR family CARD domain-containing protein 4 (NLRC4)	NLRC4 is a canonical inflammasome in NLR family that indirectly recognizes flagellin and T3SS proteins through NAIPs (22).
Noncanonical inflammasomes	Noncanonical inflammasome caspases (human caspase-4/5 and mouse caspase-11) act as both the sensor and effector, recognize stimuli such as intracellular LPS, and induce pyroptosis (6).
Nucleotide-binding domain (NBD) or NATCH	NBD is a domain in NLRs associated with ATP-induced oligomerized assembly (14).
Nucleotide-binding oligomerization domain-like receptors (NLRs)	Twenty-three and 34 NLRs have been identified in humans and mice, respectively. NLRs usually possess a LRR domain at the C-terminal and a NBD or NACHT domain in the central region (14).
Nucleotide-binding oligomerization domain-like receptor protein 3 (NLRP3)	NLRP3 is a canonical inflammasome in NLR family. Due to a lack of constitutive expression in most resting cells, activation of NLRP3 inflammasome usually requires two steps: the first signal for priming and the second signal for oligomerization and further recruitment of other components (32).
Pathogen-associated molecular patterns (PAMPs)	PAMPs are associated with pathogens and microorganism components (e.g., lipoteichoic acid [LTA] and lipopolysaccharide [LPS]) (5).
PKC-related serine/threonine-protein kinase N1 (PKN1) and N2 (PKN2)	PKN1 and PKN2 can phosphorylate pyrin, leading to interaction of pyrin with inhibitory 14-3-3 protein and maintaining pyrin in an inactive state. RhoA inhibition decreases the activity of PKN1 and PKN2 and consequently reduces the level of pyrin phosphorylation, resulting in pyrin release from 14-3-3 and accelerating pyrin inflammasome activation (33).
Pyrin domain (PYD)	PYD indirectly binds to caspase-1 *via* the homotypic interaction of PYD-PYD and CARD-CARD *via* ASC (23).
Toll-like receptors (TLRs)	TLRs are transmembrane pattern recognition receptors. TLR-, NOD ligand-, or inflammatory cytokine-mediated NF-κB-dependent transcriptional signaling provides the first signal for NLRP3 priming (5).

ATP, adenosine triphosphate; T3SS, type III secretion system.

NLR family members, such as NLRPs, NLRCs, and NAIPs, usually possess a leucine-rich repeat (LRR) domain at the C-terminal and a nucleotide-binding domain (NBD) or NACHT domain in the central region, except for NLRP10, which lacks an LRR domain, and NLRP1, which has an NACHT-LRR-C-terminal arrangement (9). LRRs contribute to ligand recognition and post-translational modifications, whereas NACHT is associated with adenosine triphosphate (ATP)-induced oligomerized assembly (14). Despite LRRs and NACHT, NLR family members usually have N-terminal domains that are responsible for caspase recruitment. CARD in NLRP1 (in the C-terminal) and NLRC4 directly binds to its counterpart domain in caspase-1, whereas PYD in other NLRP proteins, such as NLRP3, indirectly binds to caspase-1 *via* the homotypic interaction of PYD-PYD and CARD-CARD *via* the adaptor protein termed apoptosis-associated speck-like protein containing a CARD (ASC, containing a C-terminal CARD and an N-terminal PYD) (23). In contrast to other NLRs, NAIPs have three baculovirus inhibitor-of-apoptosis repeats at the N-terminus, and recognition of flagellin by NAIP5 and NAIP6 as well as recognition of type III secretion system (T3SS) rod proteins by NAIP2 indirectly activate caspase-1 through NLRC4 oligomerization (22). Once the full-length caspase-1 (containing an N-terminal CARD, a large central catalytic domain [p20], and a C-terminal, small catalytic domain [p10]) is recruited to the oligomerized inflammasome, it is activated through dimerization and autoproteolysis, and the active caspase-1 then cleaves pro-IL-1β and pro-IL-18 into their active forms. Similar activation is observed in ALR inflammasomes, which contain a C-terminal pyrin and HIN-200 domain for auto-inhibition and recognition and an N-terminal PYD for recruitment of caspase-1 with the help of ASC (26). Caspase-8 may also be involved in inflammasome activation and IL-1β production downstream of Toll-like receptors (TLRs) and Fas death receptors (34, 35). In addition, following inflammasome activation, mature caspase-1 cleaves gasdermin D (GSDMD) to its N-terminal form (GSDMD-N), which interacts with the inner membrane glycerophospholipids of the lipid bilayer, forming pores on cell membranes and triggering a lytic form of regulated cell death known as pyroptosis (29). Interestingly, GSDMD-N may also interact with cardiolipin in the bacterial membrane and the inner leaflet of the mitochondrial membrane, killing bacteria and causing mitochondrial permeabilization (36, 37). Pyroptotic pores also allow for the release of cytosolic contents, including IL-1β, IL-18, and other danger signals (38). Of note, robust production of mature IL-1β and IL-18 by inflammasomes containing CARDs may still require the involvement of ASC, whereas induction of pyroptosis may not (9, 39). Hence, activation of canonical inflammasomes elicits at least two major events: 1) maturation and release of IL-1β and IL-18 and 2) induction of pyroptosis. These events may amplify pro-inflammatory responses and contribute to tissue damage.

**Figure 1:**
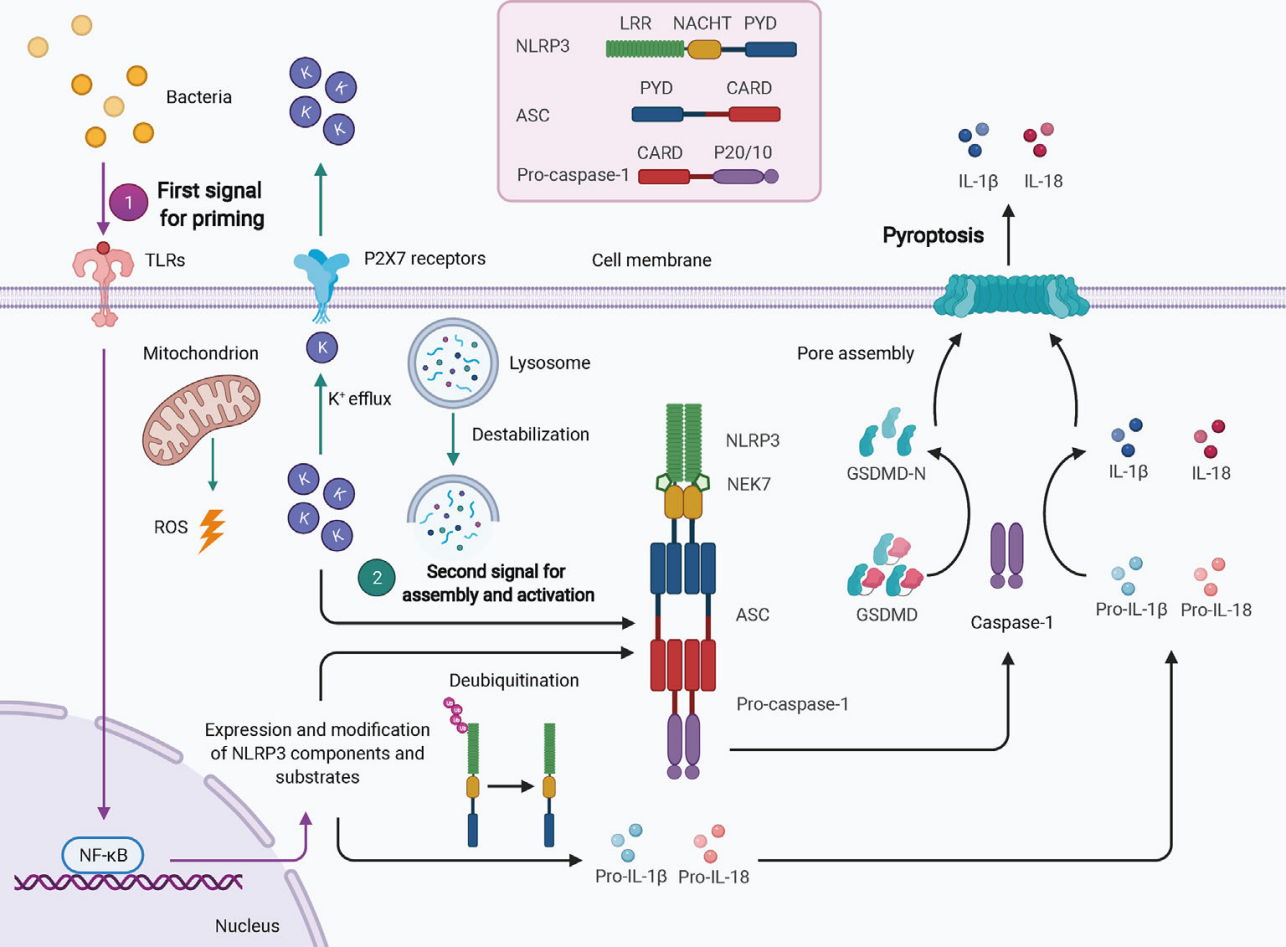
Schematic of NLRP3 inflammasome activation. In most cell types, activation of the NLRP3 inflammasome typically requires two signals. The first signal for priming (purple arrows) may result from TLR-mediated NF-κB-dependent transcriptional signaling, leading to increased expression and post-translational modification of NLRP3 inflammasome components and substrates. The second signal (green arrows) comes from a plethora of stimuli and agonists, such as ROS, potassium efflux, and lysosomal destabilization, which converge to increase cellular stress. NLRP3 oligomerizes and interacts with pro-caspase-1 with the help of ASC *via* homotypic interactions of PYD-PYD and CARD-CARD. The activated caspase-1 processes pro-IL-1β and pro-IL-18 to mature IL-1β and IL-18 and cleaves GSDMD to GSDMD-N, which inserts into the membranes to form pores, thereby leading to pyroptosis. ASC, apoptosis-associated speck-like protein containing a CARD; CARD, caspase activation and recruitment domain; GSDMD, gasdermin D; LRR, leucine-rich repeat; NEK7, NIMA-related kinase 7; NF-κB, nuclear factor-κB; PYD, pyrin domain; ROS, reactive oxygen species.

Twenty-three and 34 NLRs have been identified in humans and mice, respectively; however, only a few of these assemble into inflammasomes, such as NLRP3, NLRP1, NLRP6, NLRC4, NLRP7, and NLRP12. NLRP3 is the best-characterized inflammasome in the NLR family. Nevertheless, due to a lack of constitutive expression in most resting cells, activation of NLRP3 inflammasome usually requires two steps: the first signal for priming and the second signal for oligomerization and further recruitment of other components (**Figure 1**) (32). TLR-, NOD ligand-, or inflammatory cytokine-mediated NF-κB-dependent transcriptional signaling provides the first signal for NLRP3 priming, leading to an increase in transcriptional and translational expression of NLRP3 inflammasome components and subsequent post-translational modifications, such as phosphorylation and ubiquitination (40). Once primed, the NLRP3 inflammasome can be activated by a plethora of stimuli and agonists, including (but not limited to) infection by bacteria, viruses, and fungi, crystalline or particulate matter, reactive oxygen species (ROS) generated by ATP signaling *via* the P2X7 receptor, calcium influx, potassium efflux, chloride efflux, mitochondrial damage, oxidized mitochondrial DNA, and lysosomal destabilization, as reviewed elsewhere (41). These different agonists may converge into similar downstream events that increase cell stress as the second signal, leading to the assembly and eventual activation of NLRP3, which requires the interaction of its LRR and NBD with NIMA-related kinase 7 (NEK7) (31). The exact mechanism of NLRP3 activation is still unclear.

Other members of the NLR family also play important roles in host defense. Humans encode a single *NLRP1* gene, whereas mice harbor three multiple paralogs, namely *NLRP1A*, *-B*, and *-C*. The activation of the NLRP1 inflammasome occurs in a proteasome-dependent manner, referred to as functional degradation (42). As previously described, NLRP1 has a C-terminal CARD. Compared to other NLRs, it also has a function-to-find domain (FIIND) containing the conserved in UNC5, PIDD, and ankyrin domain (UPA) and the found in ZO-1 and UNC5 domains (ZU5) (25). Therefore, FIIND autoprocessing yields two polypeptides: UPA-CARD and NACHT-LRR-ZU5. The lethal toxin of *Bacillus anthracis* may induce the cleavage of the N-terminal of NLRP1, resulting in proteasomal N-terminal degradation (43). FIIND protects the C-terminal from degradation, and the released UPA-CARDs then undergo self-assembly for subsequent caspase-1 recruitment (44). However, whether other pathogens, such as *Toxoplasma gondii* and *Listeria monocytogenes*, induce NLRP1 activation through functional degradation remains to be elucidated. In addition, NLRP6 forms an inflammasome in response to both microbial infections and steady-state conditions (45). Lipoteichoic acid (LTA) of gram-positive bacteria, such as *L. monocytogenes*, increases NLRP6 expression and the activation of caspase-11 and caspase-1 by regulating type I IFN signaling (46). Co-expression of NLRP6 and ASC causes NF-κB activation, while NLRP6 may also negatively regulate canonical NF-κB-dependent inflammatory signaling after TLR ligation in response to *L. monocytogenes* infection (47, 48). Another NLR family member, NLRC4, indirectly recognizes flagellin through NAIP5 and NAIP6 as well as T3SS inner rod proteins through NAIP2, thereby reacting against infection by gram-negative bacteria, such as *Salmonella typhimurium* and *Legionella pneumophila* (22). However, humans express only one NAIP, which recognizes the T3SS needle protein of bacteria such as *Chromobacterium violaceum*. In addition, during bacterial infection, transcription of NAIPs is modulated by the IFN regulatory factor 8 transcription factor (49). Hence, NLRC4 inflammasome activation requires NAIPs as upstream sensors for cytosolic PAMP recognition. In addition, NOD1 and NOD2, which were among the first NLRs described, recognize bacterial peptidoglycan components and perturbations of cellular processes. They subsequently recruit the CARD-containing kinase RIP-2 *via* CARD-CARD interactions, resulting in NODosome formation and NF-κB activation (50).

**Figure 2:**
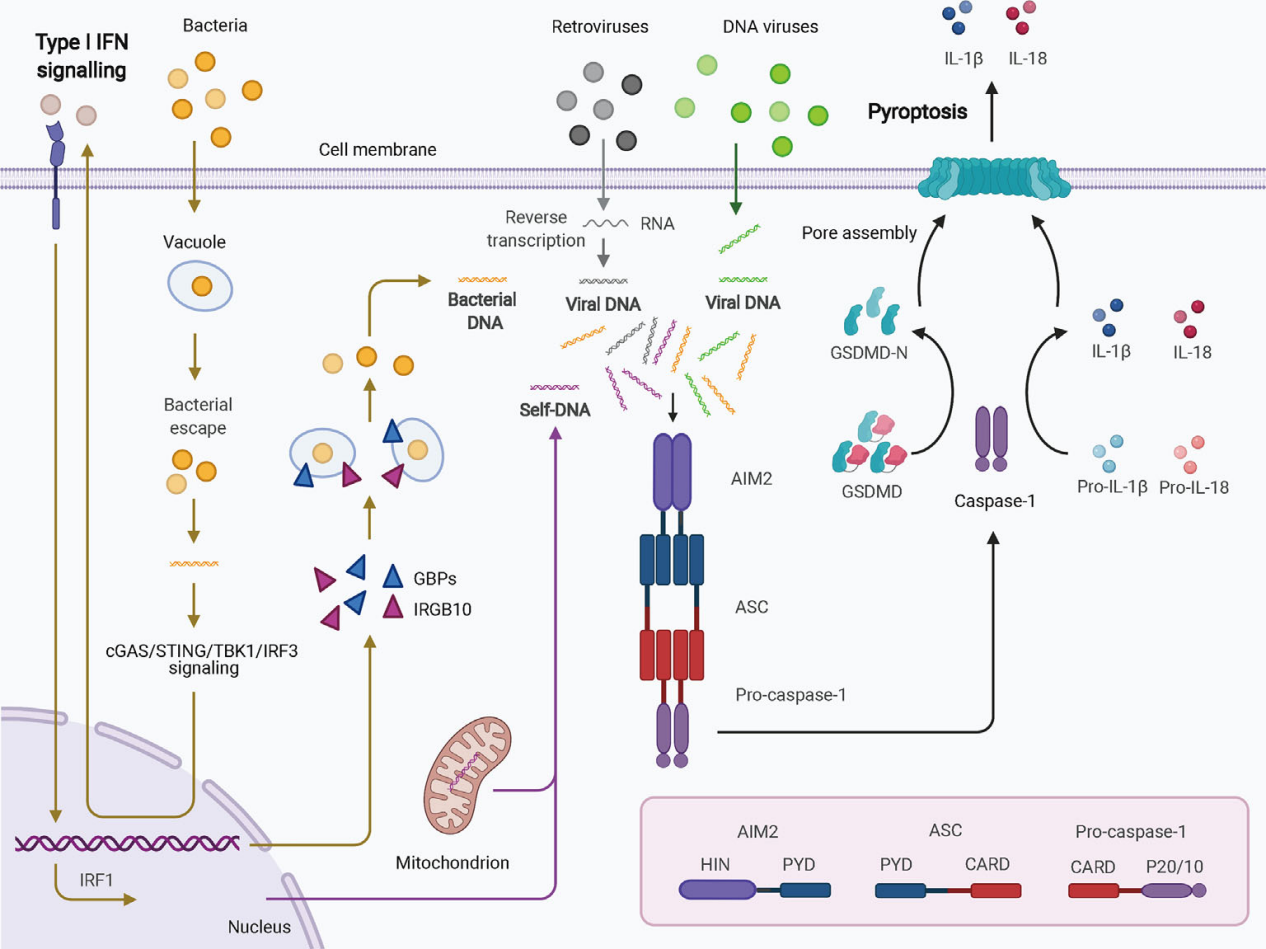
Schematic of AIM2 inflammasome activation. Cytosolic DNA from virus and self-origin directly causes “canonical” activation of the AIM2 inflammasome (arrows in grey for retroviruses, green for DNA viruses, and purple for self-DNA). However, bacteria-induced “noncanonical” activation of the AIM2 inflammasome is dependent on type I IFN signaling (yellow arrows). In this scenario, small amounts of DNA may be released from intracellular bacteria that escape from vacuoles; this DNA can be detected by cGAS. Through cGAS/STING/TBK1/IRF3 signaling, type I IFN drives IRF1 expression in an autocrine manner. GBPs and IRGB10 are then produced and disrupt the bacterial membrane and vacuoles containing bacteria, leading to exposure of a mass of DNA. DNA is then recognized by the AIM2 inflammasome. AIM2 oligomerizes and interacts with pro-caspase-1 with the help of ASC. The activated caspase-1 processes pro-IL-1b and pro-IL-18 into mature IL-1b and IL-18 and cleaves GSDMD to induce pyroptosis. AIM2, absent in melanoma 2; ASC, apoptosis-associated speck-like protein containing a CARD; CARD, caspase activation and recruitment domain; cGAS, cyclic GMP-AMP synthase; GBPs, guanylate-binding proteins; GSDMD, gasdermin D; HIN, hematopoietic interferon-inducible nuclear domain; IFN, interferon; IRF1, IFN regulatory factor 1; IRF3, IFN regulatory factor 3; IRGB10, immunity-related GTPase family member b10; PYD, pyrin domain; STING, stimulator of IFN genes; TBK1, TANK binding kinase 1.

Four and 13 ALRs are expressed in humans and mice, respectively; only AIM2 and human IFN-gamma inducible 16 (IFI16) function as inflammasomes for the recognition of cytoplasmic and nuclear DNA from pathogens and damaged cells. AIM2, first identified as a novel gene lacking in melanoma cell lines using subtractive cDNA selection and later found to be the first cytosolic member of the ALR family for innate immune sensing, recognizes double-stranded DNA (dsDNA) in a sequence-independent manner (**Figure 2**) (19–21). During bacterial infections, such as infection by *Porphyromonas gingivalis*, AIM2 inflammasome activation usually requires an upstream signal, such as type I IFN signaling. The precise mechanism of AIM2 recognition remains unclear. One proposed hypothesis suggests that, in response to bacterial infection, type I IFN is synthesized and drives the expression of IFN regulatory factor 1 (IRF1) *via* an autocrine pathway through activation of the cyclic GMP-AMP synthase (cGAS)/stimulator of IFN genes (STING)/STING-TANK binding kinase 1 (TBK1)/IRF3 axis (27). Upon IRF1 expression, guanylate-binding protein 2 (GBP2)/GBP5 and immunity-related GTPase family member b10 (IRGB10) are produced and disrupt the bacterial membrane and vacuoles containing bacteria (26, 28). Therefore, a large quantity of dsDNA is exposed and sensed by the AIM2 inflammasome. Caspase-1 activation and GSDMD-mediated pyroptosis seem to inhibit the STING pathway (51). In addition, in the context of infection by DNA viruses, such as human papillomavirus, the AIM inflammasome may recognize dsDNA directly and rapidly without the activity of type I IFN (52). IFI16, which is located in the nucleus and has two HIN-200 domains, forms an inflammasome upon infection by viruses such as herpesviruses (30). IFI16 may upregulate AIM2 expression during priming or may inhibit AIM2 inflammasome activation by impeding cytoplasmic dsDNA sensing and functional AIM2-ASC interactions (53). IFI16 also promotes p53-mediated apoptosis (54, 55). Together, canonical ALR inflammasomes are mainly responsible for dsDNA sensing and activate caspase-1 with or without the activation of type I IFN.

Pyrin, also known as TRIM20, is another canonical inflammasome that recruits caspase-1 *via* ASC, leading to IL-1β and IL-18 processing and pyroptosis. Pyrin has an N-terminal PYD (the domain is named after the protein) for ASC binding, a linker region for 14-3-3 dimer binding, a B-box domain, and a coiled-coil domain for interaction of proline-serine-threonine phosphatase-interacting protein 1 (PSTPIP1), which is critical for organization of the cytoskeleton (56). Human pyrin contains a B30.2 domain in the C-terminal, whereas murine pyrin possesses a short amino acid sequence following the coiled-coil domain. Pyrin senses pathogen-induced inhibition of the Ras homologous protein guanosine triphosphates (Rho GTPases) (57). Bacterial proteins, such as toxin B of *Clostridium difficile* and TecA of *Burkholderia cenocepacia*, decrease the activity of Ras homolog family member A (RhoA), which is a small Rho GTPase, and activate the pyrin inflammasome (58, 59). Notably, pyrin recognizes the signals downstream of RhoA modifications rather than specific modifications. Therefore, actin cytoskeletal dynamics regulated by Rho GTPases and affected by pathogen invasion may be involved in pyrin inflammasome activation (60). More precisely, the RhoA-dependent and protein kinase C-related serine/threonine-protein kinases PKN1 and PKN2 phosphorylate pyrin, leading to interaction of pyrin with inhibitory 14-3-3 protein and maintaining pyrin in an inactive state. RhoA inhibition decreases the activity of PKN1 and PKN2 and consequently reduces the level of pyrin phosphorylation, resulting in pyrin release from 14-3-3 and accelerating pyrin inflammasome activation (33). Inappropriate pyrin inflammasome activation plays critical roles in autoinflammatory diseases, such as familial Mediterranean fever (FMF), which is characterized by increased IL-1 synthesis and recurrent fever with inflammation, as mutations in *MEFV*, which encodes pyrin, are observed in FMF (61, 62). These mutations may reduce pyrin affinity to PKN1/PKN2 and disrupt the autoinhibitory state of pyrin, thus leading to constitutive pyrin inflammasome activation (60). Mutations in *PSTPIP1* may cause pyoderma gangrenosum and acne syndrome (PAPA); the PAPA-associated mutations A230T, E250Q, and E250K may increase PSTPIP1 phosphorylation, which further activates the pyrin inflammasome by increasing ASC-mediated inflammasome assembly (63–65). Therefore, the pyrin inflammasome plays a role in the pathogenesis of autoinflammatory diseases associated with mutations in the genes encoding its components. However, pyrin may inhibit IL-1β secretion by interacting with NLRP3, NLRP1, and caspase-1 and act as a negative regulator of the inflammasome signaling pathway (66–69). These functional discrepancies of pyrin in inflammasome activity remain to be clarified.

Unlike the PAMPs and DAMPs that activate the canonical inflammasome through multiprotein scaffolds, LPS may activate caspase-11 and caspase-4/5 *via* direct interactions between lipid A of LPS and CARD of caspase, resulting in the oligomerization of LPS–caspase complexes and the activation of noncanonical inflammasomes (70). GBPs and IRGB10 also contribute to this process by causing bacteriolysis *via* attack of the membranes of pathogens containing vacuoles, outer membrane vesicles (OMVs) containing LPS, and bacteria themselves; these proteins may also function as LPS receptors for recruitment of noncanonical inflammasome caspases (71). Bacterial escape into the cytosol and LPS internalization by endocytosis may cause activation of noncanonical inflammasomes without the assistance of GBPs (72, 73). Secretoglobin 3A2 may also help deliver LPS for caspase-11 activation (74). In addition to LPS, host factors, such as oxidized 1-palmitoyl-2-arachidonoyl-sn-glycero-3-phosphocholine, and pathogenic components of parasites, such as glycolipid lipophosphoglycan, may activate caspase-11 (75, 76). When activated, reminiscent of caspase-1, the noncanonical inflammasome caspases convert GSDMD into GSDMD-N to induce pyroptosis. In contrast to caspase-1, caspase-11 is not able to process pro-IL-1β and pro-IL-18, while caspase-4 may process pro-IL-18 but not pro-IL-1β, and caspase-5 possesses a weak ability to process pro-IL-1β and pro-IL-18 (24, 77, 78). These results suggest that noncanonical inflammasomes play a more important role in the induction of pyroptosis than in the direct maturation of IL-1β and IL-18. However, the increased cellular stress induced by noncanonical inflammasome activation *via* potassium efflux may trigger secondary activation of the NLRP3 inflammasome and caspase-1, thereby increasing the secretion of IL-1β and IL-18 (79, 80). This may be regarded as noncanonical NLRP3 inflammasome activation. The AIM2 inflammasome is also involved in this process in response to *L. pneumophila* infection (81). Therefore, the crosstalk between canonical and noncanonical inflammasomes increases the complexity and effectiveness of host defense against infection.

Collectively, in response to PAMPs and DAMPs, canonical inflammasomes are assembled to activate caspase-1, produce mature IL-1β and IL-18, and induce pyroptosis. Noncanonical caspases interact with stimuli, such as LPS, and trigger pyroptosis, and their crosstalk with canonical inflammasomes may cause robust secretion of IL-1β and IL-18. Pathological inactivation of inflammasomes may lead to persistent infection, whereas inappropriate activation may result in a pro-inflammatory microenvironment and excessive cell lysis. This may elicit dysregulation of host defense against PAMPs and DAMPs, in which bone destruction is implicated.

## Mechanisms of Bone Loss Related to Inflammasomes

In alveolar bone and other skeletal bone tissues, osteoclasts and osteoblasts are vital players in the delicate balance between bone resorption and formation regulated by systemic and local factors, such as cytokines and hormones (82, 83). Inflammasome activation may regulate the activities of osteoclasts, osteoblasts, and other cell types, including periodontal ligament cells, macrophages, monocytes, neutrophils, and Th17 cells, promoting a reduction in bone mass and quality, as reviewed later. Bone matrix-derived DAMPs related to osteolysis can trigger attenuated bone loss in *Nlrp3*-deficient mice compared to that in wild-type mice, and inhibition of bone resorption decreases inflammasome activation (84). Hence, inflammasome activation may be not only a promotor but also a consequence of inflammatory bone loss, indicating its role in the positive feedback mechanism of amplified inflammatory bone destruction. In this section, we focus on the mechanism of inflammasomes in bone loss, particularly in the unbalanced interplay between osteoclasts and osteoblasts and the pro-inflammatory effects on other bone remodeling-associated cells.

### Inflammasomes and Osteoclasts

Osteoclasts act as the main player in bone resorption. The receptor activator of NF-κB (RANK) ligand (RANKL)/RANK/osteoprotegerin (OPG) axis plays a pivotal role in osteoclastogenesis. Binding of RANKL to its receptor RANK recruits tumor necrosis factor (TNF) receptor-associated factor-6 and activates nuclear factor of activated T cells (NFATc1), which is the master transcription factor for osteoclast differentiation. Hence, immature myeloid progenitors differentiate into giant, multinucleated osteoclasts to resorb bone tissue properly to maintain healthy bone turnover in physiological conditions and cause excessive bone loss in pathological states. OPG, a decoy receptor of RANKL with a higher affinity than RANK, negatively regulates osteoclastogenesis. RANKL production in cells, such as osteoblasts, osteocytes, and activated T cells, can be induced by numerous factors, including prostaglandin E2 (PGE2), parathyroid hormone, progesterone, IL-17, TNF-α, and vitamin D, whereas OPG production can be induced by IL-4, estrogen, and transforming growth factor beta (85). The RANKL/RANK/OPG axis may also be regulated by B cells and T cells (86). Besides, macrophage colony-stimulating factor (M-CSF) promotes the formation of macrophage colony-forming units from hematopoietic stem cells, which expands the reservoir of common precursors of osteoclasts and macrophages (87). In addition, TLR activation at different osteoclastogenesis stages may lead to distinct outcomes. TLR2, TLR4, and TLR9 activation arrest osteoclast differentiation in progenitors stimulated with RANKL and M-CSF and maintain the cells at the macrophage stage (88). However, these TLR agonists, together with M-CSF but not RANKL, enhance osteoclastogenesis in progenitors primed with M-CSF/RANKL (89). In synergy with RANKL, TLR-induced production of TNF-α and IL-6 may also promote functional osteoclast differentiation (90, 91). When mature osteoclasts are ready to function, increased size and multinucleation of osteoclasts and polarized organization of the cytoskeleton facilitate their transportation to the microenvironment, where they produce protons (H^+^) (for mineral dissolution), cathepsin K, and collagenase (for organic component degradation) using lysosome-derived vesicles from the cytosol to resorptive sites (92, 93). RANKL and M-CSF may also play important roles in cytoskeletal reorganization, thereby promoting bone resorption (94). Therefore, the magnitude of bone resorption depends on the number of mature osteoclasts and their bone resorption capacity. In addition to their direct roles in bone degradation, osteoclasts can also act as antigen-presenting cells to modulate immune responses, as they induce regulatory T cells to inhibit osteoclast differentiation and create a negative feedback loop in the physiological state but induce TNF-α-producing CD4^+^ T cells to stimulate osteoclastogenesis under inflammatory conditions (95).

Inflammasome activation can contribute to bone resorption by regulating osteoclast activity. Engineered mice with hyperactive *Nlrp3* (D301N) specifically in osteoclasts or myeloid cells exhibited increased osteolysis compared to controls (96). PAMPs and DAMPs can activate inflammasomes in osteoclasts and pre-osteoclasts, prompting hypermultinucleation and IL-1β production (12, 97). More precisely, inflammasome activation promotes osteoclast activity by increasing osteoclastogenesis and bone resorption ability in two ways: IL-1β and IL-18 maturation; and effects of signals upstream of cytokine processing during inflammasome activation. Unlike TNF-α, IFNs, and IL-6, pro-IL-1β contains no N-terminal signal peptide for secretion and must be processed into its mature form, IL-1β. Although mast cell chymase and neutrophil proteinase 3 may also cleave IL-1β, this process is mainly regulated by inflammasome activation, as stated above (98, 99). IL-1β promotes osteoclast differentiation both directly and indirectly (100). IL-1β can induce the proliferation and multinucleation of osteoclasts derived from early blasts, myeloid blasts, and monocytes at different rates in the presence of M-CSF and RANKL (101). IL-1 can promote functional osteoclast differentiation synergistically with TNF-α (102). IL-1β also increases RANKL production in osteocytes and osteoblasts, promoting osteoclastogenesis (103–105). Moreover, IL-1β elevates M-CSF levels and decreases OPG levels (106, 107). IL-1β-triggered chemokines, such as CX3CL1, in osteoblasts regulate osteoclast precursor migration and differentiation (108). Besides promoting osteoclastogenesis, IL-1β can upregulate the expression of cathepsin K and matrix metalloproteinases (MMPs) in periodontal tissue, thereby increasing the capacity of extracellular matrix degradation of osteoclasts (109, 110). However, IL-18 may simulate or inhibit osteoclastogenesis in different cell types (111, 112). Besides their role in innate immunity, IL-1β and IL-18 may increase osteoclastogenesis by promoting B cell activation and T cell differentiation (7). IL-1β and IL-18 may stimulate RANKL production in B cells and T cells (113–115). IL-1β is required for the stable differentiation of Th17 cells from naïve T cells, and subsequent IL-17 production may stimulate RANKL and RANK production in osteoclastogenesis-supporting cells (113, 116–118). Dendritic cells, as professional antigen-presenting cells that can activate naïve T lymphocytes, may also differentiate into osteoclasts at the early development stage, and IL-1β may increase the fusion of dendritic cells into osteoclasts (119, 120). Inflammatory osteoclasts derived from dendritic cells produce higher IL-1β compared to steady-state osteoclasts derived from monocytes, which further induces TNFα-producing CD4^+^ T cells and promotes bone resorption (121). IFN-γ induced by IL-18 from activated T helper 1 cells and natural killer cells has both direct anti-osteoclastogenic and indirect pro-osteoclastogenic effects and may promote bone resorption under inflammatory conditions (122). Besides the role of IL-1β and IL-18, upstream signals during inflammasome activation also play an important role in increasing osteoclast activity. The increased NF-κB activity that acts as the first signal for inflammasome activation can increase NFATc1 transcription and promote osteoclast differentiation (123). NLRP3 signaling can cause degradation of ADP-ribosyltransferase diphtheria toxin-like 1, which disrupts its inhibitory effect on NF-κB signaling and acts as a prerequisite for osteoclast maturation (124). A hyperactive NLRP3 inflammasome can enhance osteoclast bone resorption ability by reorganizing the actin cytoskeleton (96). Together, inflammasome activation positively regulates osteoclast activity and promotes bone resorption.

### Inflammasomes and Macrophages

In addition to osteoclasts, bone marrow macrophages (hematopoietic stem cell niche macrophages, erythroblastic island macrophages) and osteal macrophages (also known as osteomacs; TRAP^–^ and F4/80^+^ macrophages) are also bone-resident macrophages (125). These macrophages can regulate bone metabolism through their communication with osteoblasts, osteoclasts, osteocytes, and mesenchymal stem cells (125). Inflammasome activation in macrophages may also promote bone destruction. NLRP3 inflammasome activation in bone marrow-derived macrophages (BMDMs) infected with *P. gingivalis* or treated with zoledronic acid increases IL-1β production (126, 127). The released IL-1β and IL-18 can then recruit more macrophages to phagocytose cell debris and kill pathogens by enhancing phagosome acidification, thereby amplifying inflammatory responses and bone resorption (99). In addition to these bone-resident macrophages that may first sense most danger-related stimuli in the local environment, macrophages derived from circulating mononuclear cells may also be recruited to infected bone tissue (128). These macrophages may be activated into pro-inflammatory M1-like macrophages or anti-inflammatory M2-like macrophages, both of which are characterized by cytokine secretion patterns and functional capabilities. Prolonged M1-like polarization usually causes chronic inflammatory conditions and tissue damage due to increased levels of ROS and pro-inflammatory IL-1β, IL-1α, TNF-α, IL-6, IL-12, IL-23, cyclooxygenase-2 (COX-2), and inducible nitric oxide synthase as well as low levels of anti-inflammatory IL-10 (129). The M1-like polarization of macrophages can be dependent on inflammasome activation, creating a pro-inflammatory milieu that is more susceptible to bone resorption (130, 131). M1-like macrophages may also promote osteocyte apoptosis in the femoral heads of a mouse model (132). In addition, inflammasome-dependent pyroptosis of cells, including macrophages, may also cause exposure of intracellular bacteria and DAMPs, facilitating their clearance in a protective way, but in some scenarios it aggravates bone inflammation (133). Hence, inflammasome activation in bone-resident and circulating macrophages upregulates the levels of IL-1β and IL-18 and promotes M1-like macrophage polarization and pyroptosis, thereby contributing to inflammatory bone loss.

### Inflammasomes and Neutrophils

Polymorphonuclear neutrophils, which are also generated from hematopoietic precursors in bone marrow and enter circulation, from which they may be recruited into infected tissues when stimulated, are also responsible for creating a pro-inflammatory environment in bone loss. Neutrophils neutralize pathogens by secreting ROS and releasing proteases and toxic enzymes *via* degranulation, killing pathogens in phagosomes *via* phagocytosis and trapping them by using neutrophil extracellular traps (NETs). Neutrophils have protective functions to maintain homeostasis and resolve inflammation by secreting anti-inflammatory resolvins and sequestering pro-inflammatory factors. However, their hyperactive actions triggered by infection or injury may also cause tissue destruction with massive upregulation of pro-inflammatory cytokines in the circulation and tissue (134). Although neutrophils have a short life span due to apoptosis, their continuous replacement ensures robust capacity in host resistance against invading microorganisms and in tissue destruction, such as alveolar bone loss in periodontitis (2). Inflammasome activation is observed in monocytes and neutrophils, which may increase antimicrobial and pro-inflammatory abilities and promote bone destruction (135). IL-1β-mediated IL-17 production can promote granulopoiesis and neutrophil release from the bone marrow *via* granulocyte colony-stimulating factor (136). IL-1R signaling is involved in infection- and inflammation-triggered emergency granulopoiesis (137). IL-1β can also induce neutrophil recruitment by upregulating the production of chemokines from other cells, such as fibroblasts, and promoting their ability to kill pathogens by increasing NET formation, degranulation, and phagocytosis (138–140). NET overproduction may trigger NLRP3 inflammasome activation in macrophages, and the increased expression of NLRP3, caspase-1, ASC, and IL-1β can be downregulated by NET digestion using DNase I (141). Proteinase 3 in neutrophils may also process pro-IL-1β in a caspase-1-independent manner (142). Hence, inflammasomes play a key role in increasing the bactericidal and pro-inflammatory ability of neutrophils, and activated neutrophils can further promote inflammasome activation.

### Inflammasomes and Osteoblasts

In contrast to the osteoclasts derived from the hematopoietic/monocyte lineage, osteoblasts, which originate from the mesenchymal/mesodermal lineage, release critical components, such as collagen fibers, osteocalcin (OCN), and osteonectin, for bone deposition and mineralization. Other osteogenic markers, such as alkaline phosphatase (ALP), runt-related transcription factor 2 (RUNX2), and osterix, are also expressed during osteoblast differentiation. After mineralization of the newly formed osteoid, osteoblasts are trapped in the bone matrix and become osteocytes, which are the most numerous cells in mature bone. Osteocytes then contact each other, osteoblasts, and osteoclasts, forming a network and demonstrating bone turnover (86). Appropriate inflammasome activity is critical in bone healing and new bone formation, as the ASC knockout mice with tibia defect exhibit delayed osteoblast differentiation compared with their wild-type counterparts (8). When inflammasomes are activated, osteoblasts promote osteoclastogenesis *via* increased production of cytokines and chemokines, such as RANKL and CX3CL1, or decreased OPG levels, as described above (107). IL-1β also affects osteoblast arrangement (143). However, production of IL-1β and IL-18 proteins in bacteria-infected osteoblasts remains controversial (144, 145). Besides the effects of IL-1β and IL-18, inflammasome activation in osteoblasts can downregulate the expression of osteogenic factors, such as ALP, RUNX2, and OCN (146). Moreover, bone marrow mesenchymal stem cells (BMSCs), which can differentiate into many cell types (e.g., osteoblasts, chondrocytes, and adipocytes in bone), also upregulate the expression of NLRP3, ASC, caspase-1, IL-1β, and TNF-α in response to LPS, thereby affecting the potential for osteogenic differentiation (147). Furthermore, Wnt/β-catenin signaling, which participates in osteoblast proliferation, differentiation, and apoptosis, may regulate NLRP3 inflammasome activity in osteoblasts; indeed, the Wnt/β-catenin pathway inhibitor Dickkopf-related protein 1 (DKK1) reversed the decreased expression of NLRP3 and active IL-1β in osteoblasts (148). Hence, increased DKK1 levels generated by osteocytes in osteolytic diseases, such as periodontitis, may also contribute to the increased activity of the NLRP3 inflammasome in these scenarios, although stronger, direct evidence is still needed (1). In addition, pyroptosis triggered by inflammasome activation can also determine the death of osteoblasts, thereby decreasing the rate of osteogenesis (149). Inhibition of NLRP3 and caspase-1 reverses the reduction in bone formation induced by pyroptosis in MC3T3-E1 cells (150). Therefore, inflammasome activation can upregulate the pro-osteoclastogenesis capacity of osteoblasts and downregulate osteoblast activity by decreasing the bone formation ability, differentiation, and proliferation of osteoblasts and promoting osteoblast pyroptosis, thereby enhancing bone resorption and decreasing new bone formation.

### Inflammasomes and Periodontal Ligament Cells

Periodontal ligament cells, mainly fibroblasts, are also involved in alveolar bone diseases, such as apical periodontitis, periodontitis, and orthodontic tooth movement (OTM) (151). Cytokines induced by inflammasome activation in periodontal ligament cells may regulate bone remodeling. IL-1β-stimulated upregulation of PGE2 expression in periodontal ligament cells increases RANKL production and promotes osteoclastogenesis (152). IL-18 upregulates the mRNA and protein levels of MMP1, MMP2, MMP3, and MMP9 in periodontal ligament cells, which may increase the resorption activity of osteoclasts (153). Inflammatory responses and/or pyroptosis also contribute to periodontium inflammation associated with alveolar bone loss (154–156). However, whether and how inflammasomes play a role in osteoclasts and osteoblasts in the periodontal ligament on the surface of the lamina dura and in the endosteal surfaces of the alveolar bone is poorly understood.

**Figure 3:**
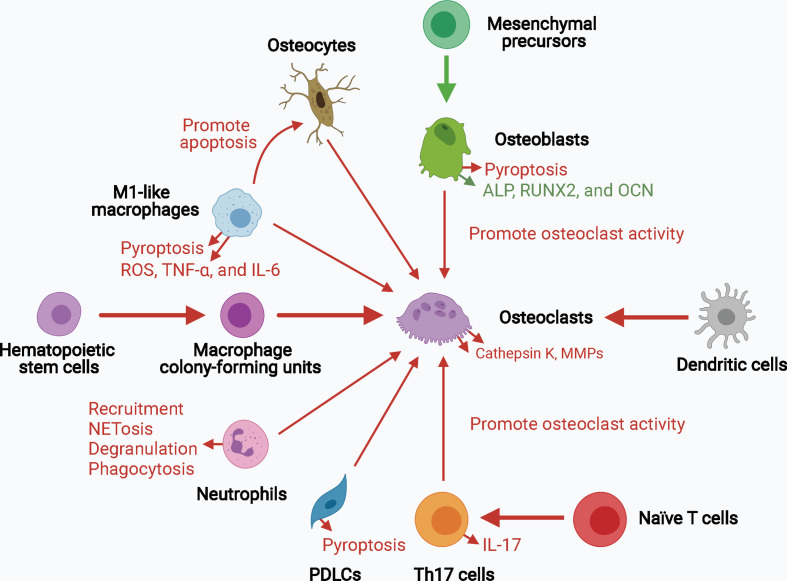
The role of inflammasome activation in the crosstalk of bone remodeling factors. Inflammasome activation promotes osteoclast activity by upregulating their differentiation from hematopoietic stem cells and dendritic cells, and enhancing their bone resorption ability. Osteoblasts, osteocytes, macrophages, neutrophils, PDLCs, and Th17 cells can also promote osteoclast activity in the context of inflammasome activation. The decreased osteogenesis and increased pyroptosis of osteoblasts and periodontal ligament cells downregulate bone formation and upregulates periodontium inflammation. Upregulated processes associated with increased inflammasome activation are marked in red, and downregulated processes are marked in green. ALP, alkaline phosphatase; MMPs, matrix metalloproteinases; OCN, osteocalcin; PDLCs, periodontal ligament cells; ROS, reactive oxygen species; RUNX2, runt-related transcription factor 2; Th17 cells, T helper 17 cells.

Collectively, inflammasome activation promotes osteoclast activity by upregulating the levels of mature IL-1β and IL-18 and signals upstream of cytokine processing during inflammasome activation. Inflammasome activation and consequent pyroptosis impair osteoblast activity and increase the destruction of periodontium. Inflammasome activation in other bone-resident and circulating macrophages, monocytes, neutrophils, and adaptive immune cells, such as Th17 cells, can contribute to creating a pro-inflammatory environment for bone destruction. Hence, inflammasome activation influences the capacity and crosstalk of bone remodeling players, particularly osteoclasts and osteoblasts, leading to increased bone degradation and decreased bone formation, thereby resulting in inflammatory bone loss (**Figure 3**).

## Inflammasomes in Inflammatory Osteolysis of the Alveolar Bone and Jaws

As stated above, inflammasomes have both protective and detrimental effects on host defense and bone remodeling. In this section, we focus on the roles of inflammasomes in the pathogenesis and development of diseases related to dysregulated alveolar bone remodeling, provide an update of current knowledge of the possible effects of inflammasome activity on different cell types (including but not limited to osteoclasts, osteoblasts, macrophages, monocytes, and periodontal ligament cells) in etiologically diverse diseases, and summarize current research gaps and potential developments in the field (**Table 2**). Moreover, given that the alveolar bone is part of the maxilla and mandible bone tissue and may be affected by pathological factors influencing the jaws, we describe several diseases associated with inflammatory bone loss in the upper and lower jaws presented as local osteolysis or a typical part of systemic osteolysis.

**Table 2:** Inflammasomes in inflammatory osteolysis of the alveolar bone and jaws.

Diseases or conditions associated with alveolar bone loss	Commonly reported PAMPs and/or DAMPs	Commonly reported inflammasome activities	Main mechanisms related to inflammasome activation in alveolar bone loss
Periodontitis	*P. gingivalis*, *F. nucleatum*, *A. actinomycetemcomitans*, and dental calculus	NLRP3 (157–162), AIM2 (163, 164), pyrin (165), and noncanonical inflammasomes (166–169) can be activated in a context- and cell type-dependent way. NLRP1 (170–174), NLRP2 (172, 175), NLRP6 (172, 176), and NLRC4 (163, 171) expressions gain controversial results. NOD1 and NOD2 may also participate in periodontitis (177, 178)	Increased osteoclast activity (179), M1-like macrophage polarization (180, 181), periodontium inflammation (155), pyroptosis of osteoblasts (149), macrophages (166, 181), and gingival fibroblasts (176, 182), as well as decreased osteogenesis (149)
Periapical periodontitis	*E. faecalis* and *P. gingivalis*	NLRP3 (135, 183), AIM2 (183), NLRP6 (184), and noncanonical inflammasomes (185) can be activated	Affected activities of osteoclasts (186), macrophages (183, 185), neutrophils (135), and periodontal ligament cells (154, 184), decreased osteoblast differentiation (146), and increased osteoblast pyroptosis (187)
Peri-implantitis	Biofilms, and release of metal ions and particles from implants	NLRP3 inflammasome can be activated (147, 188)	Increased osteoclastogenesis and M1-like macrophage polarization (189)
OTM	Factors related to external mechanical force in orthodontic treatment	NLRP3 (156, 190), NLRP1 (190), and noncanonical inflammasomes (190) can be activated	Increased osteoclastogenesis (191), M1-like macrophage polarization (156), and periodontal ligament cell pyroptosis (190, 192)
MRONJ	Antiresorptives such as zoledronic acid	NLRP3 inflammasome can be activated (127, 131)	Increased M1-like macrophage polarization (131) and macrophage pyroptosis (193)
iOM	*S. aureus* and its by-products	NLRP3 inflammasome can be activated (194, 195)	Increased activity of neutrophils (196), monocytes (195), macrophages (194, 195), and osteoclasts (150), decreased osteoblast activity (150), and enhanced osteoblast pyroptosis (150)
CNO/CRMO	Unclear	NLRP3 (197, 198) and pyrin (63) inflammasomes can be activated	Imbalanced cytokine expression (199, 200) and increased activities of osteoclasts (201), PBMCs (197), and monocytes (199, 202)
Osteoporosis	Factors related to estrogen deficiency	NLRP3 inflammasome can be activated (148, 203, 204)	Increased osteoclast activity (205), enhanced osteoblast pyroptosis (106), and decreased osteogenesis (204, 205)

CNO, chronic nonbacterial osteomyelitis; CRMO, chronic recurrent multifocal osteomyelitis; iOM, infectious osteomyelitis; MRONJ, medication-related osteonecrosis of the jaw.

### Inflammasomes in Periodontitis

Periodontitis, with an estimated 796 million severe cases globally in 2017, is a common oral inflammatory condition that induces periodontal bone loss and consequential tooth loss and acts as a risk factor for systemic disorders, such as cardiovascular disease and colorectal cancer, owing to the presence of bacteremia and inflammation (206–208). *P. gingivalis* and *Fusobacterium nucleatum* are frequently detected pathogens in chronic periodontitis, as is *Aggregatibacter actinomycetemcomitans* in localized aggressive periodontitis (133, 209, 210). IL-1β is a vital player in the pathogenesis and development of periodontitis (211). *IL1B* gene polymorphisms are a risk factor for periodontitis (212). Upregulation of IL-1β expression, which can be reversed by nonsurgical periodontal therapy, is frequently observed in saliva, periodontal pocket, gingival crevicular fluid (GCF), and serum in patients with periodontitis and is related to various clinical parameters, such as bleeding on probing (BOP) and radiographic assessment (213–215). After periodontal treatment, increased mRNA levels of *IL1B* in chronic periodontitis were also significantly reduced in peripheral blood mononuclear cells (PBMCs) of patients with BOP ≥ 16% but not of patients with BOP < 16% (216). Moreover, increased salivary IL-1β levels may have applications as a biomarker for evaluation of periodontal health in patients with type 2 diabetes and coronary heart disease (217, 218). Severe acute respiratory syndrome coronavirus may also aggravate periodontal pocket formation by enhancing the expression of pro-inflammatory cytokines, including IL-1β, and the dissemination of periodontal pathogens, and increased IL-1β release may exacerbate coronavirus disease 2019 lung infection (219, 220). Likewise, *IL18* polymorphisms are associated with susceptibility to periodontitis, and increased IL-18 levels in chronic periodontitis may positively correlate with periodontal destruction (153, 221–223).

Along with increased IL-1β processing, the activation of inflammasomes, such as NLRP3 and AIM2, is frequently detected in chronic periodontitis and aggressive periodontitis (170, 224). Negative inflammasome regulators, such as POP1, POP2, and CARD18, are also downregulated in periodontitis, further indicating increased inflammasome activity (225). Polymorphisms in various inflammasome components, including NLRP3, AIM2, and IFI16, may be associated with susceptibility to periodontitis (226–229). The mRNA levels of murine *Ifi204*, which has a structure and function similar to those of human *IFI16*, were also increased in gingival tissues of ligature-induced periodontitis (230). Salivary concentrations of ASC and NLRP3 may act as indicators of periodontal damage in periodontitis (231). ASC silencing decreases the mRNA levels of *PGE2* and its processing enzyme COX-2 in periodontitis, which may reduce bone loss (232). The authors also found that ASC-mediated PGE2 levels were increased in progressing periodontal lesions but decreased in chronic periodontitis. Among these inflammasomes, NLRP3 has been widely studied in periodontitis. NLRP3 inflammasome activation in inflammatory periodontal tissue can be triggered by local factors such as the crystalline structure of dental calculus, aggravated by systemic factors such as hyperglycemic status of type 2 diabetes mellitus and age-related oxidative stress, or alleviated by 1,25-dihydroxyvitamin D3 (157–162). NLRP3 inflammasome activation is also required for the synergistic effects of periodontal pathogens and cholesterol crystals on promoting IL-1β secretion in PBMCs, suggesting its role in the interplay between periodontal disease and cardiovascular disease (233). NLRP3 expression in GCF and periodontal parameters were increased in patients with chronic periodontitis compared to those in healthy individuals but then decreased after 6 months of combined periodontal-orthodontic treatment (234). Knockout of the *Nlrp3* gene or treatment with an NLRP3 inhibitor significantly reduces the number and differentiation of osteoclasts, thereby decreasing alveolar bone loss in mice with ligature-induced periodontitis (179). Periodontal pathogens and their by-products can also trigger inflammatory responses associated with NLRP3 inflammasome signaling (180, 235, 236). NLRP3 inflammasome activation was detected in *P. gingivalis*-induced periodontitis, leading to upregulation of IL-1β and IL-18 and enhancement of bone resorption (237). The authors also found that *Nlrp3* knockout could significantly decrease RANKL levels and increase OPG levels, indicating the importance of NLRP3 inflammasomes in promoting osteoclastogenesis in periodontitis. Inflammasome activation in macrophages infected with *P. gingivalis* may also promote inflammatory bone destruction. In an *in vitro* study, *P. gingivalis* infection was found to increase NLRP3 expression in THP-1 macrophages and human monocytic cells (Mono-Mac-6) (163, 175). Caspase-4-dependent noncanonical NLRP3 signaling participates in the dysregulation of immuno-inflammatory responses in THP-1 macrophages infected with *P. gingivalis* (238). Activation of ROS/TXNIP/NLRP3 signaling also causes migration injury of mouse periodontal ligament fibroblasts (PDLFs) treated with LPS from *P. gingivalis*, which may contribute to periodontium inflammation (155). High-dose glucose-treated *P. gingivalis* upregulates IL-1β and NLRP3 expression in human gingival fibroblasts (239). Notably, *P. gingivalis*-induced inflammasome activation may be associated with extracellular ATP and hypoxia in gingival epithelial cells and fibroblasts (171, 240–243). However, conflicting evidence shows that *P. gingivalis* infection may also inhibit NLRP3 inflammasome activation in gingival epithelial cells and fibroblasts, resulting in the escape of these bacteria from host immune defense (244–246). *P. gingivalis* may trigger proteolysis of the NLRP3 protein in endothelial cells without ATP pretreatment or LPS stimulation (247). Although the NLRP3 inflammasome is activated in cells infected with *F. nucleatum* alone, it can be repressed by co-infection with *P. gingivalis* in macrophages owing to suppression of endocytic pathways rather than reduced expression of inflammasome components (248, 249). Therefore, the effect of *P. gingivalis* on the NLRP3 inflammasome may be context-dependent in the pathogenesis and development of periodontitis. The exact mechanism through which *P. gingivalis* suppresses inflammasome activation remains unclear.

In addition, *P. gingivalis* and *A. actinomycetemcomitans* can activate the AIM2 inflammasome and increase IL-1β levels in THP-1 macrophages (163, 164). However, in BMDMs infected with *P. gingivalis*, IL-1β production is dependent on NLRP3, but not AIM2 (126). In contrast, *A. actinomycetemcomitans* infection in THP-1 macrophages can trigger robust expression of AIM2 rather than NLRP3, suggesting that activation of the AIM2 inflammasome may dominantly contribute to the defense against *A. actinomycetemcomitans* (164). Similar evidence shows that although NLRP3 expression is increased in RAW 264 cells infected with *A. actinomycetemcomitans*, it may not be the most vital player in promoting inflammatory bone loss in this scenario: inhibition of ROS and cathepsin B rather than *Nlrp3* knockdown can prevent increased IL-1β secretion, and the bone resorption activity of osteoclasts differentiated from *Nlrp3*-deficient macrophages of mice with experimental periodontitis induced by *A. actinomycetemcomitans* is even increased (250, 251). However, recent studies have shown that *A. actinomycetemcomitans* and its cytolethal distending toxin induce caspase-1 cleavage and persistent expression of IL-1β and IL-18 *via* an NRLP3-dependent pathway in U937 macrophages and THP-1 macrophages by increasing ROS and ATP levels, but not in human gingival epithelial cells (252, 253). *A. actinomycetemcomitans* may also upregulate NLRP3 expression in mononuclear leukocytes, without affecting the level of AIM2 (172). Moreover, the salivary concentration of NLRP3 is higher in patients with aggressive periodontitis than in those with chronic periodontitis (231). Furthermore, *Nlrc4*-knockout mice exhibit greater bone resorption than wild-type mice, and osteoclast activity is increased in *Nlrc4*-deficient macrophages, suggesting a protective role of NLRC4 inflammasomes in inflammatory bone resorption in periodontitis induced by *A. actinomycetemcomitans*, which may be attributed to NLRP3 inflammasome activation (254). Therefore, whether NLRP3 or AIM2 inflammasomes are more predominant in *A. actinomycetemcomitans*-induced periodontitis remains unclear. A possible explanation is that *A. actinomycetemcomitans* may differentially activate inflammasome signaling pathways in the host cells of periodontal tissues. These conflicting results support the complexity of the effects of inflammasomes in the pathogenesis of periodontitis.

In addition to NLRP3 and AIM2, the effects of other canonical inflammasomes on the pathogenesis and development of periodontitis have also been investigated. NLRP1 inflammasomes do not show significant activation in *P. gingivalis*-infected gingival epithelial cells, *A. actinomycetemcomitans*-infected mononuclear leukocytes, and gingival fibroblasts exposed to 6-species supragingival or 10-species subgingival biofilms (171–173). However, NLRP1 expression is significantly increased in human periodontal ligament cells (hPDLCs) challenged by advanced glycation end-products by activating the NF‐κB pathway, supporting its role in the influence of diabetes on periodontitis (174). In contrast, NLRP1 levels are decreased in the gingival tissues of mice with ligature-induced periodontitis (170). The NLRP2 inflammasome is not activated in *A. actinomycetemcomitans-*infected mononuclear leukocytes (172). However, *NLRP2* mRNA levels are increased in gingival tissues from patients with chronic periodontitis and generalized aggressive periodontitis but reduced in Mono-Mac-6 cells infected with *P. gingivalis* (175). Furthermore, *A. actinomycetemcomitans* downregulates NLRP6 expression in mononuclear leukocytes (172). As described above, NLRC4 may have protective roles in *A. actinomycetemcomitans*-induced periodontitis; however, *P. gingivalis* infection was shown to not activate NLRC4 inflammasomes in THP-1 macrophages and gingival epithelial cells (163, 171). Additionally, NOD1 and NOD2 in the NLRC family may also participate in inflammation in periodontitis. Mice lacking *Nod1* and receiving ligature placement exhibit reduced alveolar bone resorption with decreased recruitment of neutrophils and osteoclasts, whereas mice lacking *Nod2* exhibit no differences in bone destruction compared to control mice (177). However, *Nod2* knockout was shown to decrease osteoclastogenesis and alveolar bone destruction in mouse periodontitis induced by heat-killed *A. actinomycetemcomitans*, which may be associated with the affected NLRP3 inflammasome activity (178). In addition, as stated above, mutations in *MEFV*, which encodes pyrin, are involved in FMF. Patients with FMF harboring R202Q and M694V mutations in *MEFV* present higher percentages of BOP, clinical attachment levels, mean gingival indexes, and probing pocket depths than healthy controls (165). *TRIM20* mRNA levels are also downregulated in gingival tissues of patients with gingivitis, chronic periodontitis, and aggressive periodontitis compared to those in healthy controls (225). These results suggest a possible role of pyrin in the development of periodontal disease.

Inflammasome-induced pyroptosis may also cause dysregulated bone remodeling and aggravated tissue inflammation in periodontitis (255). Oxidative stress induces pyroptosis of osteoblast-like MG63 cells by activating the NLRP3 inflammasome, thereby attenuating bone formation and promoting periodontitis; in contrast, an NLRP3 inhibitor reverses the reduction in osteoblast migration and COL1, RUNX2, and ALP levels (149). *P. gingivalis* activates the double-stranded RNA (dsRNA)-dependent kinase in osteoblastic MC3T3-E1 cells, thereby promoting NLRP3 expression by activating NF-κB, and LPS from *P. gingivalis* triggers NLRP3 inflammasome-dependent pyroptosis of gingival fibroblasts, which can be alleviated by eldecalcitol (a vitamin D analog) and inhibitors of ROS or NLRP3 (256, 257). *P. gingivalis* and its LPS may also induce pyroptosis of gingival fibroblasts by activating NLRP6 and NLRP3 (176, 182). *A. actinomycetemcomitans* infection also triggers the death of osteoblast-like MG63 cells *via* activation of the NLRP3 inflammasome, and leukotoxin from this bacterium may induce pyroptosis of macrophages in a P2X7 receptor-mediated and NLRP3-dependent manner (145, 258, 259). Pyroptosis induced by NEK7-dependent NLRP3 inflammasome activation is also critical in diabetes-associated periodontitis (31). Hence, inflammasome-dependent pyroptosis plays an essential role in alveolar bone loss in periodontitis. In addition, there may be differences between bacteria and their by-products with regard to their effects on metabolic remodeling and pyroptosis in macrophages. Both *P. gingivalis* and its OMVs trigger the reprogramming of metabolic gene expression and M1-like macrophage polarization in murine macrophages (181). Moreover, *P. gingivalis* OMVs induce inflammasome complex formation in 80% of macrophages *in vivo* (180). However, in one study, OMVs from *P. gingivalis* were found to increase lactate dehydrogenase (LDH) release from macrophages, indicating the occurrence of pyroptosis induced by inflammasome activation, whereas *P. gingivalis* alone did not promote LDH release; in another study, *P. gingivalis* was found to increase GSDMD-N expression and induce pyroptosis in macrophages (166, 181). In addition, *P. gingivalis* can increase noncanonical caspase-11/4 expression in macrophages (166, 167). *Treponema denticola* and *Tannerella forsythia* activate caspase-1 and caspase-4 and trigger pyroptosis in THP-1 macrophages (168). Td92, a surface protein of *T. denticola*, and Tp92, a homolog of *Treponema pallidum* surface protein, trigger caspase-4-dependent pyroptosis in human gingival fibroblasts *via* activation of cathepsin G (169). Td92 can also activate the NLRP3 inflammasome *via* ATP release and potassium efflux (260). These data suggest a role for noncanonical inflammasome activation in periodontitis.

Collectively, both canonical and noncanonical inflammasome activation contribute to alveolar bone loss in periodontitis, and these processes may be affected by systemic factors. More precisely, increased osteoclast activity, M1-like macrophage polarization, periodontium inflammation, and pyroptosis of osteoblasts, macrophages, and gingival fibroblasts as well as decreased osteogenesis may be involved in these processes. Differences can be detected between different pathogens and between pathogens and their by-products with regard to effects on inflammasome activity, and these differences may be context- and cell type-dependent. Further investigations are required to obtain a comprehensive understanding of the roles and mechanism of inflammasomes in the pathogenesis and development of periodontitis.

### Inflammasomes in Periapical Periodontitis

Periapical periodontitis, a common oral disorder with a reported prevalence of 5% at the tooth level and 52% at the individual level, is primarily characterized by infection of root canals and inflammatory periapical tissues, including the periodontal ligament and alveolar bone (261). *Enterococcus faecalis* and *P. gingivalis* are commonly detected pathogens in infected root canals and apical root surfaces of periapical periodontitis, respectively (183, 262). IL-1β may play vital roles in bone loss in periapical periodontitis: *IL1B* gene polymorphisms are involved in the risk of periapical periodontitis development (263); IL-1β production is increased in periapical periodontitis *in vivo* (264); and IL-1β levels and osteoclast differentiation are upregulated in an *in vitro* coculture system of osteoblasts and osteoclasts (186). However, the roles of IL-18 in the pathogenesis and development of periapical periodontitis remain unclear.

Increased IL-1β production in periapical periodontitis may be associated with the activation of NLRP3 and AIM2 inflammasomes. NLRP3 expression is observed in macrophages, fibroblasts, vascular endothelial cells, monocytes, and neutrophils in diseased periapical tissue and is positively correlated with inflammatory intensity (135). The upregulated IL-1β expression in inflammatory periapical tissues and infected hPDLCs is dependent on increased NLRP3 and ASC expression, and *ASC* silencing reduces IL-1β levels (154). Additionally, NLRP6 expression was also detected in inflammatory periapical tissues and was found to negatively regulate TNF-α and IL-6 levels by inhibiting extracellular signal-regulated kinase (ERK) and NF-κB signal pathways, and knockdown of *NLRP6* in hPDLCs may increase NLRP3 expression (184). Bacteria and their by-products can promote inflammasome activation and induce periapical bone loss. LTA of *E. faecalis* increases the expression of NLRP3 and caspase-1 *via* upregulation of ROS and activation of NF-κB in RAW264.7 cells, and this effect can be reversed by inhibitors of NLRP3 or NF-κB (146, 265). Moreover, *E. faecalis* infection triggers atypical M1-like macrophage polarization in murine bone marrow-derived stem cells; however, the roles of inflammasomes in this process remain unclear (266). LPS from *P. gingivalis* increases the mRNA levels of *NLRP3*, *AIM2*, *ASC*, and caspase-1 in THP-1 macrophages (183). Noncanonical inflammasomes may also participate in the pathogenesis of periapical bone loss. The increased expression of caspase-1 and caspase-11 in RAW264.7 cells treated with LPS was found to be significantly reduced by nanosilver, and this decreased inflammasome activity may contribute to the alleviation of canine periapical periodontitis progression (185). In addition, estrogen deficiency can induce activation of the NLRP3/caspase-1/IL-1β axis and aggravate periapical bone loss in postmenopausal patients and ovariectomized rats with periapical periodontitis (267). This suggests a role for inflammasomes in the effect of systemic risk factors on the development of periapical periodontitis.

Inflammasome activation also contributes to decreased osteoblast activity in periapical periodontitis. The NLRP3 inhibitor dioscin protects osteoblast-like MC3TE-E1 cells treated with LTA from *E. faecalis* from morphological changes and reverses the downregulation of osteogenic factors, such as ALP, RUNX2, and OCN, thereby promoting mineralized nodule formation (146). Moreover, inflammasome-induced pyroptosis plays vital roles in periapical periodontitis. Along with increased expression of NLRP3, caspase-1^+^/terminal deoxynucleotidyl transferase dUTP nick end labeling^+^ cells were observed in apical inflammatory tissues of chronic periapical periodontitis, indicating the occurrence of pyroptosis. Pyroptosis was also significantly increased in rats with acute periapical periodontitis, resulting in increased bone loss, and this effect could be alleviated by caspase-1 inhibition, suggesting that pyroptosis levels may be related to the degree of inflammation in periapical periodontitis (268). *E. faecalis* increases GSDMD cleavage in THP-1 macrophages, leading to pyroptosis *via* activation of the NLRP3 inflammasome; this mechanism requires the P2X7 receptor and potassium efflux (269). *E. faecalis* also increases LDH release from MG63 cells, and this process can be blocked by treatment with a caspase-1 inhibitor or silencing of *NLRP3*, supporting the occurrence of pyroptosis in osteoblasts (187). LPS from *P. gingivalis* induces caspase-1-mediated pyroptosis in human PDLFs (268). *Candida albicans*, another species that is frequently isolated from endodontic infections of periapical periodontitis, was shown to induce pyroptosis by activating the NLRP3 inflammasome in mononuclear phagocytes and macrophages (270). Taken together, these data suggest that periapical periodontitis pathogens may induce pyroptosis to promote inflammation and bone destruction.

Collectively, canonical inflammasomes, such as NLRP3 and AIM2, and noncanonical inflammasomes may be involved in the pathogenesis and development of periapical periodontitis. In addition to osteoclasts, macrophages, and neutrophils, periodontal ligament cells may also be affected by inflammasome activation induced by pathogens and their by-products, resulting in periodontium inflammation. Inflammasome activation also attenuates osteogenesis by decreasing osteoblast differentiation and increasing osteoblast pyroptosis. Further investigations are needed to elucidate the effects of inflammasomes on macrophage polarization in periapical periodontitis.

### Inflammasomes in Peri-Implantitis

Dental implants are widely used in the treatment of edentulism. Peri-implantitis, with a prevalence ranging from 1.1% to 85% at the implant level and a higher early failure rate in maxillary implants than mandibular implants, occurs in the peri-implant region and often leads to inflammatory loss of supporting bone (188, 271, 272). Radiographic bone loss greater than or equal to 2 mm beyond the crestal bone level from the initial surgery, or greater than or equal to 3 mm apical to the most coronal part of the intraosseous portion of the implant is observed in peri-implantitis, with even greater progression than that in periodontitis (273). Similar to periodontitis, peri-implantitis exhibits higher IL-1β levels in diseased tissues, and these changes may persist despite nonsurgical therapy (274–276). Genetic polymorphisms in *IL1B* are related to the risk of peri-implantitis and contribute to increased clinical parameters, such as peri-implant pocket depth, plaque index, and clinical attachment level (277). However, the role of IL-18 in the pathogenesis of peri-implantitis remains unclear.

The pathogenesis of peri-implantitis is associated with a series of factors, including the action of biofilms, release of metal ions and particles from implants, and infiltration of inflammatory cells (e.g., polymorphonuclear leukocytes), thereby resulting in osseointegration failure and implant rejection (278–280). Pathogen invasion from the implant surface biofilm is a critical inflammatory stimulus in peri-implantitis owing to a lack of effective epithelial barriers (281). These pathogens and their by-products can trigger inflammasome activation. *Candida* spp., frequently found in peri-implantitis lesions, can induce activation of the NLRP3 inflammasome (282). LPS from *P. gingivalis*, another peri-implantitis-related pathogen, increases the mRNA levels of *NLRP3*, *ASC*, and caspase-1 in BMSCs, thereby increasing IL-1β production (147). Moreover, iron and particles from dental implants can also induce inflammasome activation (283). Particles released by titanium implants trigger an inflammatory response in preosteoclasts, promote M1-like macrophage polarization, and increase osteoclastogenesis, which can be affected by IL-1β-neutralizing antibodies (189). Titanium ions activate the NLRP3 inflammasome by increasing the production of ROS in Jurkat T cells, leading to immune responses in peri-implantitis (188). However, another study showed that titanium ions alone induced only limited mRNA levels of *NLRP3*, *ASC*, and caspase-1 in macrophages and demonstrated that IL-1β secretion could be enhanced by LPS priming (284).

Overall, these findings show that metal ions and particles from implants and pathogens induce inflammasome activation in peri-implantitis, thereby promoting alveolar bone loss mainly by increasing osteoclastogenesis and enhancing inflammation. However, the roles of noncanonical inflammasomes in peri-implantitis are poorly understood. The possible roles of inflammasome-induced pyroptosis and periodontium inflammation in the pathogenesis and development of peri-implantitis should be evaluated in further studies.

### Inflammasomes in OTM

External mechanical force in orthodontic treatment can cause stress on both the periodontal ligament and alveolar bone, leading to bone loss on the compression side and bone regeneration on the tension side; this results in OTM (285). Inflammatory bone resorption in OTM often differs from that in periapical periodontitis and periodontitis, as it is triggered by mechanical stress rather than bacterial infection (286). Inflammasome activation is important in alveolar bone loss during OTM. IL-1β is frequently detected in the GCF during OTM, and its production can be upregulated by increasing the orthodontic force (287–289). The expression of IL-1β and RANKL was found to be increased in patients undergoing orthodontic treatment using injectable platelet-rich fibrin, whereas that of OPG was found to be significantly decreased, indicating the promotion of osteoclastogenesis (191). The levels of NLRP3, caspase-1, and IL-1β were shown to be increased in the periodontium tissues of rats subjected to excessive orthodontic force, and activation of the NLRP3/caspase-1/IL-1β axis as well as polarization of M1-like macrophages was also detected in THP-1 cells when cocultured with force-pretreated hPDLCs but inhibited by the NLRP3 inhibitor MCC950 (156). In response to cyclic stretching, the levels of NLRP3, NLRP1, cleaved caspase-1, cleaved caspase-5, cleaved GSDMD, IL-1β, and IL-18 were shown to be increased in hPDLCs, leading to pyroptosis; this process was partly blocked by treatment with a caspase-1 inhibitor or knockdown of *GSDMD* (190, 192). These data suggest that inflammasome activation is involved in alveolar bone loss in the context of orthodontic mechanical force, which is closely associated with periodontium inflammation. However, cyclic stretching may suppress NLRP3 inflammasome activation and IL-1β secretion in macrophages by inhibiting the activity of caspase-1 rather than NF-κB (290, 291). Exosomes from hPDLCs stimulated with cyclic stretching suppress IL-1β production in macrophages by inhibiting the NF-κB signaling pathway (292). In addition, as current studies are mostly focused on NLRP3 inflammasomes in OTM, further studies are needed to assess the possible roles of other forms of inflammasomes, including noncanonical inflammasomes, in alveolar bone loss associated with OTM.

### Inflammasomes in Medication-Related Osteonecrosis of the Jaw (MRONJ)

MRONJ, which was initially reported as bisphosphonate-related osteonecrosis of the jaw (BRONJ) in 2003, is characterized by necrotic bone loss of the jaw induced by antiresorptive and anti-angiogenic drugs (293, 294). Although MRONJ can occur spontaneously, tooth extraction, prosthetic trauma, dental surgery, periodontal disease, dental implant, and periapical periodontitis may act as triggering or exacerbating factors, and patients with MRONJ may present concomitant diseases, such as diabetes mellitus or hypertension, or be administered chemotherapeutic drugs or corticosteroids (295, 296). The high turnover rate in the jaw may explain the typical localization of osteonecrosis in this region compared to other skeletal tissues (4, 294). The mechanism of MRONJ remains unclear and may be attributed to impaired bone remodeling and jaw vascularization, and increased inflammation. Bisphosphonate treatment can promote MRONJ. Although bisphosphonate exhibits antiresorptive effects on osteoclasts, this drug can also cause dysregulation of osteoblast and osteoclast coupling, eventually resulting in necrotic bone loss of the jaw (297). More specifically, the expression of ALP in osteoblasts is suppressed in MRONJ, and the acidic microenvironment also increases osteoblast inhibition and decreases new bone formation (298). Increased IL-1β levels are associated with inflammation in MRONJ, enhancing nonvital bone tissue and decreasing newly formed bone tissue (299, 300). The number of IL‐1β^+^ cells is significantly increased in rats treated with nitrogen-containing bisphosphonate zoledronic acid and subjected to left inferior molar extraction, whereas that of cells positive for IL-18-binding protein (IL-18 bp), a natural antagonist of IL-18, is increased in rats treated with 0.04 mg/kg zoledronic acid and decreased in a dose-dependent manner (301). Zoledronic acid increases the expression of IL-1β in an NLRP3/caspase-1-dependent manner in LPS-primed BMDMs from mice with diabetes mellitus, and NLRP3 inhibitors improve oral wound healing and suppress osteonecrosis of the jaw in these mice (127). Zoledronic acid triggers M1-like macrophage polarization and increases the mRNA and protein levels of IL-1β by activating the NLRP3 inflammasome and cleaving pro-caspase-1 in LPS-primed THP-1 cells; these effects can be reversed by silencing of *ASC* (131). Zoledronic acid also induces caspase-1-dependent and GSDMD-mediated pyroptosis and secretion of IL-1β in RAW264.7 cells, by mediating methylation of histone H3 (H3k27me3) (193). Furthermore, increased numbers of Th17 cells and IL-17 levels were found to be correlated with elevation of the M1/M2 macrophage ratio in human and murine BRONJ lesions (302).

Collectively, these findings show that bisphosphonates induce bone loss by activating the inflammasome. M1-like polarization and pyroptosis of macrophages may promote a pro-inflammatory environment that is prone to bone destruction. However, despite NLRP3, the roles of other canonical and noncanonical inflammasomes in the pathogenesis of MRONJ remain unclear. More information is also needed to elucidate the possible relationships between inappropriate inflammasome activity and bone loss in MRONJ induced by antiresorptive drugs.

### Inflammasomes in Nonsterile or Sterile Osteomyelitis of the Jaw

Infectious osteomyelitis (iOM) of the jaw, an entity separate from osteonecrosis of the jaw, is an infection of the bone and bone marrow that results in inflammatory bone loss and aberrant bone neoformation in the jaw (303). iOM of the long bones commonly results from hematogenous spread and local extension, whereas iOM of the jaw may arise from local infection of the oral cavity, paranasal sinuses, and skin. Immune dysfunction, metabolic abnormalities, malnourishment, alcohol consumption, and vascular insufficiency may act as risk factors for iOM (304). *Staphylococcus aureus* is the most prevalent pathogen of hematogenous and post-traumatic iOM, and *P. gingivalis* may be detected as the leading bacteria in lesions of iOM in the jaw related to periodontitis (305, 306). Evidence has shown that inflammasome activation is involved in *S. aureus*-induced iOM (307). Caspase-1 activity and IL-18 levels are upregulated in neutrophils and monocytes in the blood of patients with *S. aureus* bacteremia, which could lead to iOM, supporting the occurrence of inflammasome activation (196). Toxic shock syndrome toxin 1 from *S. aureus* and ATP significantly increase IL-1β expression through activation of TLR4 and NLRP3 in mouse peritoneal macrophages (194). Additionally, Panton-Valentine leukocidin from *S. aureus* causes the release of IL-1β and IL-18 from human monocytes and macrophages owing to the activation of NLRP3 and caspase-1 (195). These increased levels of IL-1β and IL-18 in neutrophils, monocytes, and macrophages may promote osteoclastogenesis in iOM. In addition, *S. aureus* internalization in osteoblasts contributes to the pathogenesis of iOM (308). Inflammasome activation in *S. aureus*-infected osteoblasts may decrease the intracellular replication of *S. aureus*. *S. aureus* strains defective in toxin genes encoding phenole-soluble modulins induce lower levels of IL-1β in MG63 cells compared to strains harboring a functional Agr system, and *S. aureus*-induced inflammasome activation and intracellular *S. aureus* clearance require the activation of caspase-1 (309). Similar results were observed in phagocytic cells, in which inflammasome activity is needed to limit *S. aureus* replication (310). These results suggest a positive role for inflammasome activation in host defense against *S. aureus* by limiting its replication and increasing its clearance. However, inflammasome activation also decreases osteoblast activity in the context of *S. aureus* infection. The levels of NLRP3 and GSDMD were found to be increased in infectious bone tissue from patients with osteomyelitis compared to those in bone fragments from patients with fractures; moreover, caspase-1 and NLRP3 inhibitors significantly reduce *S. aureus*-induced osteoblast pyroptosis, restore bone formative properties, and attenuate osteoclast activation in bone marrow macrophages *in vitro* and decrease bone loss *in vivo* (150). Specifically, the levels of dsRNA were found to be increased in a chicken model and in patients with osteomyelitis, and *DICER1* (encoding endoribonuclease for dsRNA cleavage) knockdown or *Staphylococcus* infection-induced dsRNA accumulation upregulates IL-1β and IL-18 expression in and reduces viability of human osteoblasts *via* activation of the NLRP3 inflammasome, indicating that DICER1 and dsRNA dysmetabolism is an upstream regulator of NLRP3 signaling in infected osteoblasts as a model of osteomyelitis (311). Taken together, these findings suggest that inflammasome activation affects the activity of neutrophils, monocytes, macrophages, osteoblasts, and osteoclasts and contributes to inflammatory bone loss in iOM.

In contrast to bacteria-induced osteomyelitis, chronic nonbacterial osteomyelitis (CNO) and the more severe multifocal form of chronic recurrent multifocal osteomyelitis (CRMO) are autoinflammatory bone disorders with recurrent clinical symptoms, such as pain, local swelling, and impairment of bone motion resulting from periosteal and/or endosteal inflammation, osteomyelitis, and osteitis (312, 313). CNO can affect any site in the skeleton, including the jaws, in all age groups, with a peak onset from 7 to 12 years of age (201). CRMO of the jaw may result in multifocal and symmetrical bony damage in the long bones as radiographically lytic or sclerotic lesions (314). Some adult patients with CRMO develop complex symptoms of synovitis, acne, pustulosis, hyperostosis, and osteitis (315). The pathogenesis of sporadic CNO/CRMO remains unclear. Increased levels of pro-inflammatory IL-1β, TNF-α, IL-6, and IL-20 and decreased levels of anti-inflammatory IL-19 and IL-10 have been observed in monocytes from patients with CNO/CRMO, and imbalances in cytokine expression may contribute to inflammatory bone loss (199, 200). Upregulation of IL-1β-mediated osteoclast differentiation and activation is associated with inflammasome activation (201). Notably, mRNA levels of caspase-1 and IL-1β were found to be significantly increased in PBMCs from patients with CRMO at active and remission stages compared to those in healthy controls, and the expression of NLRP3, ASC, caspase-1, and IL-1β was also detected in bone tissues of patients with CRMO (197). DNA methylation of *NLRP3* and *PYCARD*, which encodes ASC, was decreased in monocytes from patients with CRMO compared to that in healthy individuals, leading to increased gene expression (198). Reduced IL-19 and IL-10 expression, which may be caused by decreased ERK1 and ERK2 activities and impaired epigenetic remodeling, also enhances the activation of the NLRP3 inflammasome in CRMO monocytes, and recombinant IL-19 or IL-10 significantly reduces IL-1β levels (199, 202). In addition, three diseases associated with chronic multifocal sterile osteomyelitis that may result from single gene mutations, including deficiency of IL-1 receptor antagonist (mutations in *IL1RN* encoding the IL-1 receptor antagonist), Majeed syndrome (*LPIN2* mutations), and PAPA, also exhibit IL-1β-mediated bone inflammation, highlighting the roles of inflammasomes in their pathogenesis (316). As stated above, mutated PSTPIP1 in PAPA may increase the activity of the pyrin inflammasome, leading to increased IL-1β expression and aggravated autoinflammation (63). Bone autoinflammation in mice with CRMO resulting from *Pstpip2* gene mutation may be independent of AIM2 but can be protected by deficiencies in NLRP3/caspase-1 and caspase-8 signaling, suggesting that caspase-8 plays a role in IL-1β processing (317, 318). Hence, inappropriate inflammasome activation is critical for sterile osteomyelitis induced by gene mutations.

Collectively, these findings support the involvement of inflammasome activation in host defense against extracellular pathogens and in the recognition of endogenous DAMPs in jaw osteomyelitis. In particular, in sporadic and familial or monogenic CNO/CRMO, upregulation of pro-inflammatory cytokines and downregulation of anti-inflammatory cytokines contribute to increased osteoclast activity, in which inflammasome-dependent IL-1β acts as a vital player. Hence, IL-1 signaling regulatory agents may be a therapeutic option for CNO/CRMO treatment (319). Further studies are required to elucidate the roles and mechanisms of noncanonical inflammasomes in nonsterile or sterile osteomyelitis of the jaw.

### Inflammasomes in Osteoporosis Related to Alveolar Bone Loss

Osteoporosis is a metabolic skeletal problem characterized by dysregulation of osteoclast and osteoblast activity, leading to decreased bone density and increased bone degradation, bone fragility, and fracture risk (320). According to a 2005–2010 survey by the Division of Health and Nutrition Examination Surveys, the age-adjusted prevalence of osteoporosis at the femur neck or lumbar spine in adults aged 65 and over is 24.8% in women and 5.6% in men in the United States (321). A plethora of factors may affect bone remodeling in osteoporosis, including hormones such as estrogen and testosterone, sex, age, hyperglycemia, gut microbiome, dietary intake, and loading (322). Some patients may not be aware of osteoporosis because they experience no symptoms until fracture occurs. Therefore, early detection of osteoporosis is critical.

Bone remodeling of the maxilla and mandible, including the alveolar bone, is involved in bone turnover in the skeletal system and reflects the condition of skeletal bone. Bone loss in the jaw is involved in osteoporosis and may act as a screening predictor for osteoporosis and fracture risk. The mandibular cortical index, mandibular inferior cortical width below the mental foramen, and alveolar trabecular bone pattern of the mandible are useful for screening of low skeletal bone mineral density (BMD) and osteoporosis (323). The relative risk of future fracture of the sparse trabecular pattern of the mandible is higher than that of cortical erosion in perimenopausal and older women (324). Moreover, evaluation of trabecular bone density in the mandibular premolar region may also facilitate the detection of osteoporosis (325). Owing to the high ratio of trabecular bone to cortical bone, analysis of the trabecular bone in the maxilla may also provide a good opportunity to screen for osteoporosis (326). Additionally, the mean radiographic density in the interdental and alveolar regions in the maxilla and interdental region in the mandible was found to be lower in women with osteoporosis than in healthy controls (327). Therefore, considering the correlation between bone loss in the jaw and osteoporosis and the fact that dental X-ray examinations are easier and more convenient than skeletal BMD measuring techniques, such as dual-energy X-ray absorptiometry, it is essential to develop strategies for osteoporosis screening using dental radiographs that evaluate bone loss in the jaw. Investigating the mechanisms of osteolysis in osteoporosis may also improve our understanding of alveolar bone loss in this scenario.

Upregulation of IL-1β, enhanced osteoclastogenesis, and decreased osteogenesis are observed in osteoporosis, partly because of inflammasome activation (328, 329). In fact, mice with the humanized NLRP3 locus and disease-associated mutations develop thinner and radiolucency cortices, consistent with osteoporosis (203). The expression of NLRP3, ASC, and cleaved caspase-1 is also increased in the femoral bone of ovariectomized mice and osteoblasts derived from BMSCs of these mice, leading to increased production of IL-1β and IL-18. Additionally, knockdown of *Nlrp3* significantly upregulates RUNX2 and OCN in BMSCs of ovariectomized mice (148). IL-18 bp inhibits the activation of the NLRP3 inflammasome and increases osteoblast differentiation *in vitro* and reduces osteoclastogenesis and Th17 cell differentiation *in vivo*, thereby preserving cortical bone parameters and restoring the trabecular microarchitecture in ovariectomized mice (205). The NLRP3 inflammasome can also be activated in mesenchymal stem cells treated with LPS and palmitic acid, leading to increased adipogenic differentiation and decreased osteogenic differentiation; these effects may be blocked by caspase-1 inhibition (204). Moreover, increased osteoblast death dependent on NLRP3 expression has also been observed in rats with postmenopausal osteoporosis, leading to decreased trabecular thickness, trabecular number, trabecular separation, and BMD (330). Therefore, osteoblast pyroptosis induced by inflammasome activation may play a pivotal role in osteoporosis (106). In addition, inflammasome activation is involved in the interplay of other systemic/local diseases with bone loss in osteoporosis. High glucose conditions increase the levels of ROS, phospho-ERK, phospho-JNK, phospho-p38, NF-κB, NLRP3, ASC, caspase-1, IL-1β, and IL-18 in rat osteoclasts differentiated from bone marrow-derived monocytes, suggesting a role of inflammasome activation in the interaction between diabetes mellitus and osteoporosis (331). Moreover, estrogen deficiency may aggravate bone loss in periapical periodontitis, and osteoporosis may also promote osteolysis in periodontitis, which may involve inflammasome activation (332). Furthermore, as described above, antiresorptive bisphosphonate, which can be used for osteoporosis treatment, may also cause inflammasome-associated bone loss in the jaw. These results highlight the roles of the NLRP3 inflammasome in promoting bone loss in osteoporosis.

Collectively, inflammasome activation may upregulate osteoclast capacity and impair osteoblast activity by reducing osteogenic differentiation and increasing osteoblast death. Owing to the potential association of periodontitis/periapical periodontitis with osteoporosis and the use of bisphosphonate medication in osteoporosis, dysregulation of bone remodeling in osteoporosis is quite complex, particularly in the jaw bone and alveolar bone, in which symptoms of bone loss converge and must be carefully distinguished and analyzed. The exact roles of inflammasomes in the pathogenesis and development of osteoporosis, particularly direct evidence of alveolar bone loss using human biopsies and mouse models, need to be elucidated in subsequent studies.

## Conclusions and Perspectives

**Figure 4:**
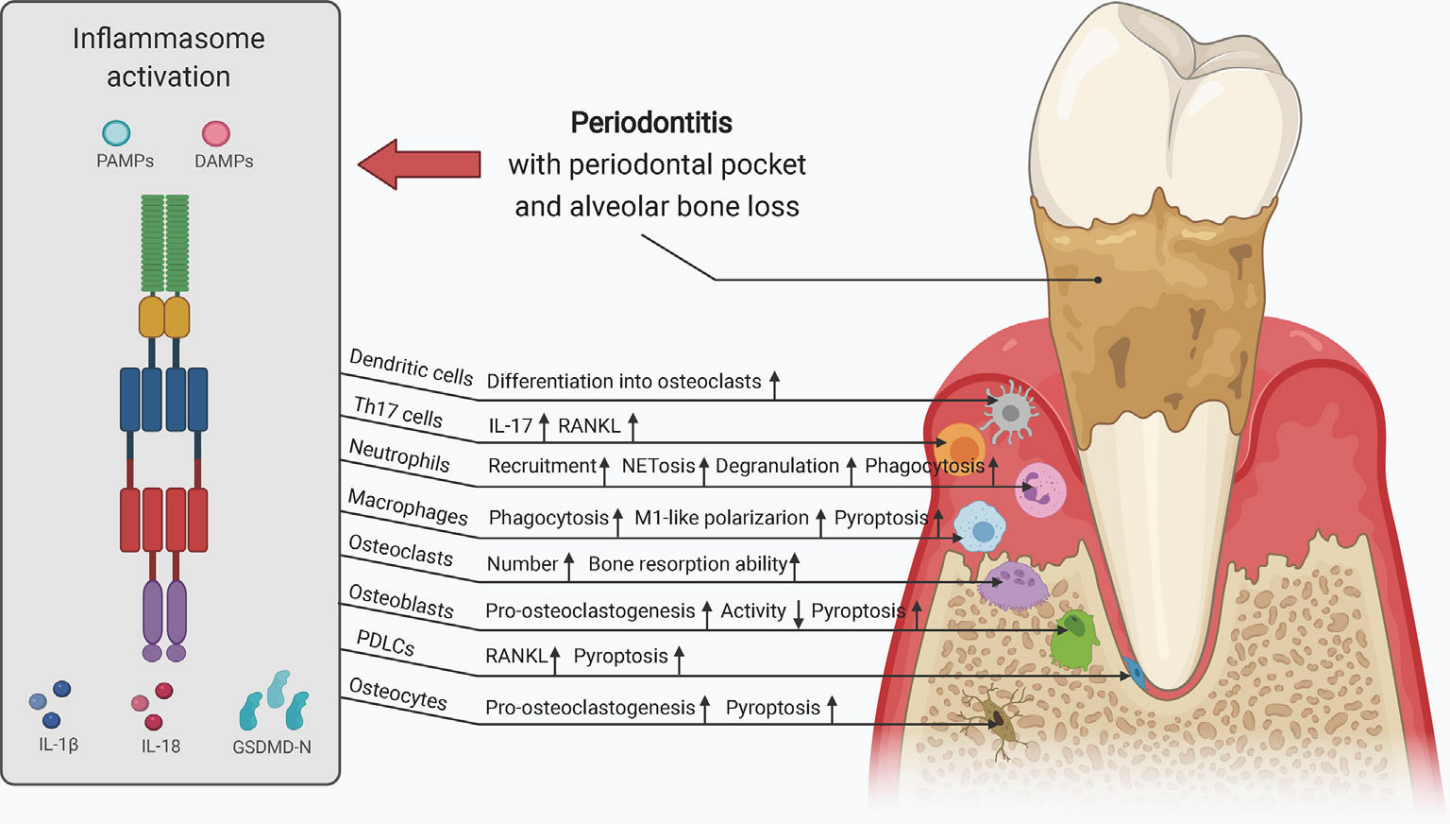
Schematic of the role of inflammasome activation in alveolar bone loss (e.g., periodontitis). Inflammasome activation is pivotal in alveolar bone loss *via* the following mechanisms: 1) increasing osteoclast activity; 2) decreasing osteoblast activity; 3) creating a pro-inflammatory milieu that facilitates bone resorption; and 4) causing periodontium inflammation by affecting periodontal ligament cells. Osteoclasts, osteoblasts, osteocytes, PDLCs, macrophages, neutrophils, T cells, and dendritic cells may be affected by inappropriately increased inflammasome activity, contributing to dysregulation of alveolar bone remodeling. DAMP, damage-associated molecular pattern; PAMP, pathogen-associated molecular pattern; PDLCs, periodontal ligament cells; RANKL, receptor activator of NF-κB ligand. Th17 cells, T helper 17 cells.

Similar to other bone tissues, alveolar bone remodeling is intricately regulated by osteoclasts and osteoblasts. A high turnover rate and associations with the tooth and periodontium highlight the increased complexity of alveolar bone remodeling. Pathogen infection, mechanical stress, medication, and systemic pathological factors are common causes of alveolar bone loss. Such features make alveolar bone a typical and important site for the investigation of the underlying mechanisms of dysregulated bone remodeling. The rapid growth of information in osteoimmunology has improve our understanding of the mechanisms of dysregulated alveolar bone remodeling. As a double-edged sword, the inflammasome exerts both protective and harmful effects on host defense and alveolar bone remodeling. Notably, excessive inflammasome activation may play a pivotal role in alveolar bone loss *via* the following mechanisms (**Figure 4**). First, it can increase osteoclast activity, e.g., by promoting osteoclastogenesis *via* decreasing OPG release or increasing RANKL levels, or increase the bone resorption capacity of osteoclasts by upregulating the expression of cathepsin K and MMPs. These effects are attributed to elevated levels of IL-1β and IL-18 and to other signals upstream of cytokine processing during inflammasome activation. Second, it can decrease osteoblast activity by reducing the bone formation ability, proliferation, and differentiation of osteoblasts and inducing osteoblast pyroptosis. Third, it can create a pro-inflammatory milieu that facilitates bone resorption by causing pyroptosis, M1-like macrophage polarization, neutrophil infiltration, and adaptive immune responses. Finally, it can cause periodontium inflammation by affecting periodontal ligament cells. Since the periodontium connects tooth and alveolar bone, and its destruction may indirectly lead to pathological effects on both of them, the mechanisms of excessive inflammasome activation in periodontal ligament cells require special attention.

However, more evidence should be collected to fully unveil the role of inflammasomes in alveolar bone loss. Although evidence has demonstrated that inflammasome activation promotes the direct bone resorption ability of osteoclasts, whether it can influence osteoclast-modulated T cell activation remains unclear. The most predominant activated or inactivated inflammasomes involved in the pathogenesis and development of specific diseases associated with alveolar bone loss still need to be clarified, as do the mechanisms through which crosstalk or interplay between different inflammasomes contribute to alveolar bone loss. Studies on these topics may also provide insights into the regulators modulating the activity of inflammasomes in alveolar bone loss. The precise mechanism of inflammasome activation in periodontium inflammation also requires further investigation. In addition, the roles of inflammasomes in other diseases associated with alveolar osteolysis, such as alveolar osteitis (dry socket) and osteoradionecrosis, remain to be elucidated. Nevertheless, current investigations of inflammasomes have provided important insights into osteoimmunology and contribute to our understanding of the cellular and molecular mechanisms of alveolar bone loss.

Based on these observations and considerations, it is plausible that novel drug-based strategies for targeting inflammasome activity may contribute to the treatment of alveolar bone loss. Accumulating evidence has suggested that excessive inflammasome activation may be mitigated by the following strategies: 1) regulation of inflammasome priming, e.g., using E and D series resolvins that reduce NF-κB activity (333); 2) regulation of upstream signaling associated with inflammasome oligomerization and activation, e.g., targeting intracellular ROS by using antioxidant drugs, such as SS-31 (also known as Bendavia or MTP-131) (334); 3) regulation of inflammasome components, e.g., targeting caspase-1 using its inhibitors, such as VX-765, targeting NLRP3 using MCC950 and β-hydroxybutyrate, or using P2X7 receptor antagonists, such as AFC-5128 and GSK1482160 (211, 335); 4) regulation of the pro-inflammatory effects of inflammasome-dependent cytokines, e.g., targeting IL-1β using a recombinant receptor antagonist (anakinra) and monoclonal antibodies (gevokizumab and canakinumab) (336); and 5) regulation of pyroptosis. Some of these therapeutic strategies have already been explored in studies on alveolar bone loss, such as periodontitis and Majeed syndrome; however, most of the relevant investigations are still in their foundational phase (230, 319). The joint application of anti-inflammasome drugs and routine therapies such as scaling and root planning for periodontitis and root canal therapy for periapical periodontitis should be considered carefully. Moreover, as most of the potential drugs are administered orally or subcutaneously, more convenient and direct modes of access during routine treatments such as drug delivery into deep periodontal pockets or into periapical lesions *via* infected root canals could be novel therapeutic strategies (230). Notably, although the combination of multiple strategies and versatile drugs may help create promising opportunities for the treatment of bone loss, much work is still needed to assess the therapeutic inhibition of inflammasomes, which should be subtly balanced with the beneficial contributions of inflammasome activation in host defense. Further studies are needed to fully elucidate the roles and mechanisms of inflammasomes in the pathogenesis, development, and treatment of alveolar bone loss.

## Author Contributions

YL wrote the manuscript and created the figures. QJ and JL reviewed and edited the manuscript and provided guidance. All authors contributed to the article and approved the submitted version.

## Funding

This work was supported by the Project of the Educational Commission of Guangdong Province of China (No. 2020KZDZX1161).

## Conflict of Interest

The authors declare that the research was conducted in the absence of any commercial or financial relationships that could be construed as a potential conflict of interest.
